# DEPDC1B is a key regulator of myoblast proliferation in mouse and man

**DOI:** 10.1111/cpr.12717

**Published:** 2019-12-11

**Authors:** Nicolas Figeac, Johanna Pruller, Isabella Hofer, Mathieu Fortier, Huascar Pedro Ortuste Quiroga, Christopher R. S. Banerji, Peter S. Zammit

**Affiliations:** ^1^ King's College London Randall Centre for Cell and Molecular Biophysics London UK

**Keywords:** cell proliferation, DEPDC1B, human myoblasts, rhabdomyosarcoma, RHOA, satellite cells

## Abstract

**Objectives:**

DISHEVELLED, EGL‐10, PLECKSTRIN (DEP) domain‐containing 1B (DEPDC1B) promotes dismantling of focal adhesions and coordinates detachment events during cell cycle progression. DEPDC1B is overexpressed in several cancers with expression inversely correlated with patient survival. Here, we analysed the role of DEPDC1B in the regulation of murine and human skeletal myogenesis.

**Materials and methods:**

Expression dynamics of DEPDC1B were examined in murine and human myoblasts and rhabdomyosarcoma cells in vitro by RT‐qPCR and/or immunolabelling. DEPDC1B function was mainly tested via siRNA‐mediated gene knockdown.

**Results:**

*DEPDC1B* was expressed in proliferating murine and human myoblasts, with expression then decreasing markedly during myogenic differentiation. SiRNA‐mediated knockdown of DEPDC1B reduced myoblast proliferation and induced entry into myogenic differentiation, with deregulation of key cell cycle regulators (cyclins, CDK, CDKi). DEPDC1B and β‐catenin co‐knockdown was unable to rescue proliferation in myoblasts, suggesting that DEPDC1B functions independently of canonical WNT signalling during myogenesis. DEPDC1B can also suppress RHOA activity in some cell types, but DEPDC1B and RHOA co‐knockdown actually had an additive effect by both further reducing proliferation and enhancing myogenic differentiation. *DEPDC1B* was expressed in human Rh30 rhabdomyosarcoma cells, where *DEPDC1B* or RHOA knockdown promoted myogenic differentiation, but without influencing proliferation.

**Conclusion:**

DEPDC1B plays a central role in myoblasts by driving proliferation and preventing precocious myogenic differentiation during skeletal myogenesis in both mouse and human.

## 1 INTRODUCTION

DISHEVELLED, EGL‐10, PLECKSTRIN (DEP) domain‐containing 1B (DEPDC1B) and its paralog DEPDC1A are cell cycle–regulating proteins.^1^ The *DEPDC1B* gene, at human chromosome 5q12, encodes a 61 kDa protein of 529 amino acids. DEPDC1B contains an N‐terminal DEP domain and a C‐terminal RHO‐GAP (GTPase‐activating protein)‐like domain. The DEP domain is a globular region discovered in DISHEVELLED, EGL‐10 and PLECKSTRIN and plays a role in mediating membrane localization,^2^ and DEPDC1B is usually membrane‐associated, being highly expressed during G2/M phase of the cell cycle.^1^, ^3^ The RHO‐GAP domain is involved in RHO GTPase signalling (eg RAC, CDC42 and RHO) that regulates cell motility, growth, differentiation, cytoskeleton reorganization and cell cycle progression.^4^ Membrane association via the DEP domain enables DEPDC1B to interact with G protein‐coupled receptors, as well as membrane phospholipids necessary for Wnt signalling. However, the GAP domain of DEPDC1B lacks the critical arginine residue required for GAP activity.^1^ The GAP domain of DEPDC1B can also interact with the nucleotide‐bound forms of RAC1 and can control their activation.^5^, ^6^

DEPDC1B can also indirectly suppress activation of RHOA.^1^ The transmembrane protein tyrosine phosphatase receptor type F (PTPRF) and the guanine nucleotide exchange factor H1 (GEF‐H1) are required for RHOA activation. DEPDC1B inactivates RHOA by competing for binding of PTPRF, so allowing cell de‐adhesion and cell cycle progression.^1^

DEPDC1B expression oscillates during cell cycle progression, accumulating at the G2 phase, similar to checkpoint proteins such as cyclin B, which correlates with its function as a regulator of cell cycle.^1^ DEPDC1B knockdown induces a significant delay in transition to mitosis, due to impairment of the de‐adhesion process.^1^ RHOA is required for formation and integrity of focal adhesion points, and DEPDC1B, as an indirect inhibitor of RHOA, promotes dismantling of focal adhesions, necessary for morphological changes preceding mitosis.

RHO GTPases including RHOA, RAC1 and CDC42 are also crucial regulators of skeletal myogenesis,^7^ and their precise temporal regulation is critical for efficient myotube formation.^7^, ^8^ RHOA is required for the initial induction of myogenesis by activating serum response factor (SRF) 9 which induces the myogenic transcription factor MyoD.^10^, ^11^, ^12^ In myocytes however, RHOA perturbs localization of M‐cadherin, a cell adhesion molecule required for myoblast fusion,^13^ and so needs to be inactivated before myoblast fusion.^14^ Such inactivation is mediated by RHOE and GRAF1.^15^, ^16^ Therefore, precise modulation of RHOA activity is required for differentiation to proceed.^17^ While Rac1 and CdC42 are required for myoblast fusion in Drosophila in vivo,^18^ overexpression of RAC1 or CDC42 inhibits myogenesis in rat myoblasts.^19^ RAC1 and CDC42 can have this dual role by activating the C‐Jun N‐terminal kinase (JNK), a negative regulator of myogenesis, but also activating the stress‐activated protein kinase (SAPK) and p38: pathways necessary for myogenesis.^20^ Moreover, RAC1 inhibits myogenic differentiation by preventing complete withdrawal of myoblasts from the cell cycle 21 and exogenous expression of RAC1 and CDC42 impair cell cycle exit and induce loss of cell contact inhibition.^22^ This suggests a function of RAC1 and CDC42 during proliferation, rather than during the differentiation process.

DEPDC1B expression is repressed by PITX2, a bicoid‐related homeobox transcription factor implicated in regulating the left‐right patterning and organogenesis.^6^, ^23^, ^24^ The first intron of the human and mouse *DEPDC1B* gene contains multiple consensus DNA‐binding sites for PITX2, and knockdown of PITX2 in murine C2C12 myoblasts promotes an increase in DEPDC1b at the protein level. PITX2 particularly, but also PITX3, are additionally involved in regulation of muscle development and adult muscle stem (satellite) cell function.^25^, ^26^, ^27^, ^28^, ^29^, ^30^, ^31^

Finally, DEPDC1B is overexpressed in various cancers including breast, oral, non‐small‐cell lung, melanoma and prostate, and represents a potential biomarker and therapeutic target.^3^, ^5^, ^32^, ^33^, ^34^, ^35^, ^36^ Interestingly, in many human cancer cells, DEPDC1B also has a nuclear location.^37^ DEPDC1B overexpression in breast cancer cells can increase phosphorylation of ERK and promote cell proliferation and delay cell death.^3^ DEPDC1B is also highly expressed in oral cancer and overexpression induces augmentation of ERK1/2 activity by RAC1 GTP, promoting cell migration and invasion in cancer cell lines.^5^ DEPDC1B up‐regulation in non‐small‐cell lung cancer has a reverse correlation with patient survival, and its overexpression promotes tumour cell migration and invasion through activating Wnt/β‐catenin signalling.^33^ Increased DEPDC1B expression in prostate cancer is associated with an advanced clinical stage of disease.^32^ Deregulation of RHO GTPases is implicated in tumours that have characteristics of skeletal muscle (rhabdomyosarcomas),^22^, ^38^, ^39^ but the role of DEPDC1B in rhabdomyosarcomas is unknown.

Here, we examined the role of DEPDC1B in both mouse and human skeletal myogenesis. *DEPDC1B* was expressed in both mouse and human proliferating myoblasts, but expression fell precipitously during myogenic differentiation. SiRNA‐mediated knockdown of DEPDC1B drastically reduced proliferation and induced precocious myogenic differentiation in both species. In myogenic cells, DEPDC1B function was unaffected by manipulation of β‐catenin, while DEPDC1B expression was unaltered by PITX2 levels. Given that DEPDC1B can suppress RHOA activation, it was unexpected that DEPDC1B/RHOA co‐knockdown had an additive effect in myoblasts, reducing proliferation and enhancing differentiation more than DEPDC1B knockdown alone. *DEPDC1B* was expressed in human Rh30 rhabdomyosarcoma cells at a higher level than in myoblasts, and while *DEPDC1B* down‐regulation did not affect proliferation, it did promote myogenic differentiation. Thus, DEPDC1B plays a central role in driving proliferation and preventing differentiation during skeletal myogenesis.

## 2 MATERIALS AND METHODS

### 2.1 Ethics statement

Mice were bred in accordance with British law under the provisions of the Animals (Scientific Procedures) Act 1986, as approved by the King's College London Ethical Review Process committee.

### 2.2 Myofibre isolation and culture of mouse satellite cells

Mice aged between 8 and 12 weeks were killed by cervical dislocation, and the extensor digitorum longus (EDL) muscles were isolated and digested as previously described.^40^ Freshly isolated myofibres were either cultured under non‐adherent conditions or plated on Matrigel, and satellite cell‐derived myoblasts were then expanded using DMEM‐GlutaMAX (Invitrogen Life Technologies), with 30% foetal bovine serum (FBS) (Gibco), 10% horse serum (Invitrogen Life Technologies), 1% chick embryo extract (MP), 10 ng/mL bFGF (PreproTech) and 1% penicillin/streptomycin (Sigma), again as previously described.^40^

### 2.3 Cell culture

Immortalized human myoblasts (C25Cl48) were originally isolated from the semitendinosus of a 25‐year‐old individual and were kindly provided by Dr Vincent Mouly (Institute Myology, Paris, France).^41^ For proliferation, C25Cl48 myoblasts were maintained in skeletal muscle cell growth medium (PromoCell, C‐23160) supplemented with 20% FBS, or switched to differentiation medium (PromoCell: C23161). Proliferation and differentiation medium were supplemented with 50 µg/mL of gentamycin. Preparation of cDNA from human primary myoblasts was described previously.^42^

Rhabdomyosarcoma Rh30^43^ and RMS‐YM cells were maintained in proliferation in DMEM‐GlutaMAX (Invitrogen Life Technologies) with 10% FBS (Gibco) and 1% penicillin‐streptomycin (Sigma). To induce differentiation, cells were cultured in growth medium until confluent and then switched to differentiation medium (PromoCell: C23161).

### 2.4 Retroviral expression vectors

The retroviral backbone pMSCV‐puro (Clontech) was modified to replace the puromycin selection gene with an *IRES‐eGFP* to create *pMSCV‐IRES‐eGFP*, which served as control.^44^ Mouse *Depdc1b‐V5* (Depdc1b‐201 transcript encoding for a protein of 529 amino acids with a V5‐Polyhistidine region at the C‐terminus) and the human *PITX2C* cDNA (transcript variant 3, coding for a protein of 324 amino acids) were amplified by RT‐PCR and cloned into *pMSCV‐IRES‐eGFP*. Retrovirus was then packaged in 293T or Phoenix cells using standard methods. Proliferating satellite cells and immortalized human myoblasts (C25Cl48) were transduced in 6‐well plates with 500 μL of retrovirus or lentivirus in 1.5 mL of proliferation medium with polybrene (4 μg/mL). Due to a low retroviral transduction rate in C25Cl48 immortalized human myoblasts, cells were expanded and GFP‐positive cells (transduced cells) FAC‐sorted, expanded for a few days and then stably transduced cells analysed.

### 2.5 siRNA‐mediated gene knockdown

Primary satellite cell‐derived myoblasts were transfected with siRNA, either Silencer Select Pre‐designed siRNA used at 8 nM final concentration with Silencer Select Negative Control, or 2 nM Qiagen FlexiTube GeneSolution siRNA (eg GS218581 for Depdc1b, four siRNAs: SI00977984, SI00977977, SI00977970, SI00977963). For immortalized human myoblasts (C25Cl48), siRNAs were from either Life Technologies or QIAGEN (FlexiTube GeneSolution, four siRNAs used individually at 2 nM final concentration with AllStars Negative Control). All siRNAs are detailed in Table S1.

Satellite cells were transfected with siRNA in DMEM‐GlutaMAX, 30% FBS, 10% horse serum and 1% chick embryo extract for 24 h at 37°C. Transfection of siRNA into immortalized human myoblasts (C25Cl48) was performed in skeletal muscle cell growth medium (PromoCell) for 24 h at 37°C. All siRNA transfections were performed using Lipofectamine RNAiMAX as per manufacturer's instructions.

### 2.6 Western blot

Primary murine satellite cells were transfected with siRNA against DEPDC1B (Si Depdc1B Mix or Si Depdc1B N2) and allowed to proliferate for 72 h and then collected for Western blot analysis. Blots were probed against DEPDC1B (Sigma, HPA072558‐100UL, 1:500), and total protein was visualized using the Bio‐Rad TGX Stain‐Free Technology. For quantification, pixel intensity of either the whole lane (total protein image) or the band corresponding to DEPDC1B (DEPDC1B immunolabelled image) was quantified using Fiji, and knockdown efficiency presented as DEPDC1B intensity normalized to whole protein intensity.

### 2.7 RT‐qPCR

Mouse or human myoblasts were cultured in 6‐well plates in proliferation medium or switched to differentiation medium for the stated duration. Total RNA was extracted using the RNeasy Kit (Qiagen) and cDNA prepared from 500 ng to 1 μg of RNA with the QuantiTect Reverse Transcription Kit with genomic DNA wipeout (QIAGEN). qPCR was performed on a Mx3005PQPCR system (Stratagene) with Brilliant II SYBR Green reagents and ROX reference dye (Stratagene) or QuantiNova SYBR Green PCR Kit (QIAGEN) using primers shown in Table S2. Relative expression between proliferating and differentiated myoblasts and between control and test samples was measured in 3 replicates and significance tested using a two‐tailed Student's t test in Microsoft Excel.

### 2.8 Immunolabelling of cells

EdU incorporation was assayed using a Click‐iT EdU Imaging Kit (Life Technologies) as per manufacturer's instructions. Satellite cell‐derived myoblasts were immunolabelled as previously described.^45^ Briefly, fixed myoblasts were permeabilized with 0.5% Triton X‐100/PBS for 10 min at room temperature and blocked with 5% goat serum/PBS for 60 min at room temperature. Primary antibodies (Table S3) were applied overnight at 4°C, washed and visualized with fluorochrome‐conjugated secondary antibodies (Invitrogen) used at 1/500 for 1 h at room temperature. Preparations were then incubated in DAPI (300 µM) for 10 min and washed in PBS.

### 2.9 Image acquisition

Images of plated myoblasts were acquired on a Zeiss Axiovert 200 M microscope using a Zeiss AxioCam HRm and AxioVision software version 4.4 (Zeiss). For colocalization analysis, images were acquired on a confocal microscope (Zeiss). Images were adjusted globally for brightness and contrast and analysed with ImageJ and RStudio^46^ software.

### 2.10 Statistical testing

For analysis of immunolabelling following siRNA treatment, cells in multiple unit areas per well per experimental condition were counted, and data from each mouse were expressed as a single mean ± SD. Such mean ± SD from at least three mice were used for each condition tested. Significant difference (*P* < .05) between control and a test sample was determined using a paired two‐tailed t test in Excel (Microsoft). For clonal cell lines, cells in multiple unit areas per well per experimental condition were counted and data from each well expressed as a single mean ± SD. Such mean ± SD from at least three independent wells were used for each condition tested and significant difference (*P* < .05) between control and a test sample determined using an unpaired two‐tailed *t* test in Excel (Microsoft). For RT‐qPCR, at least three independently siRNA‐transfected wells per condition were assessed.

## 3 RESULTS

### 3.1 DEPDC1B is located in the nucleus of murine satellite cell‐derived myoblasts

**Figure 1:**
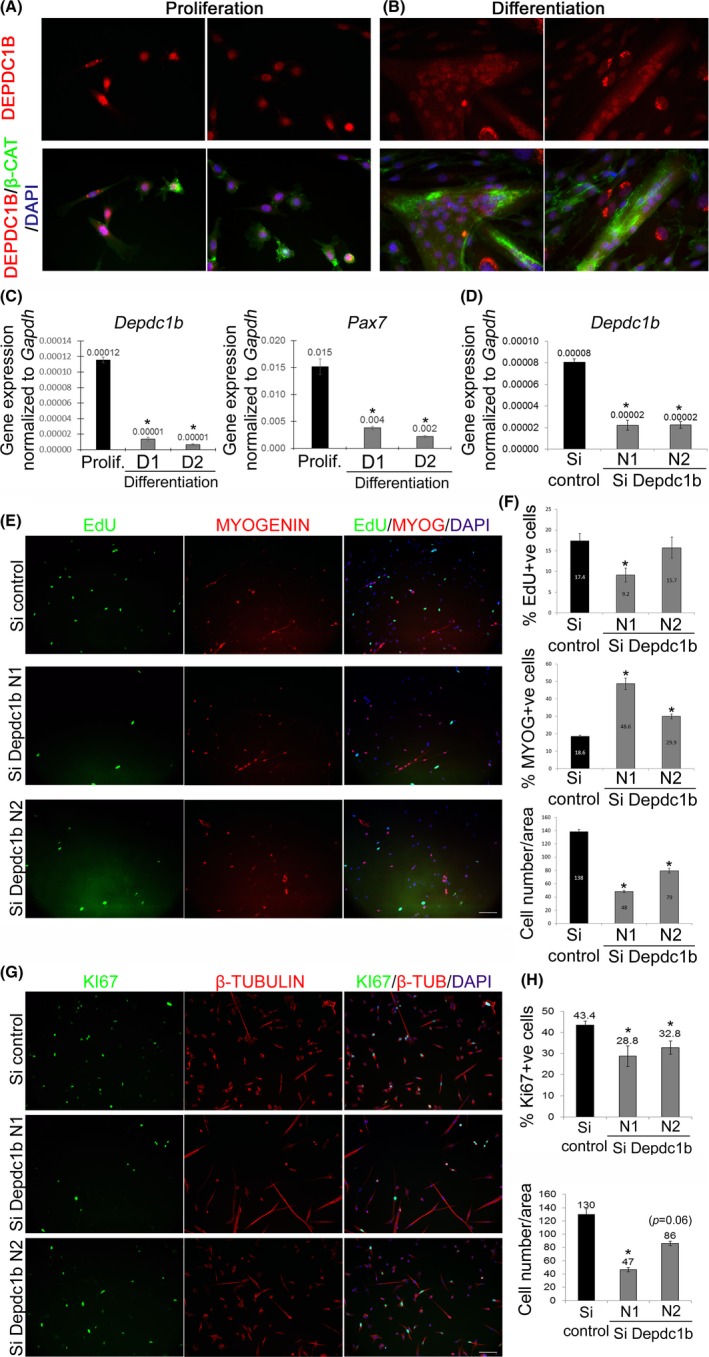
DEPDC1B controls proliferation and differentiation in mouse satellite cell‐derived myoblasts. (A and B) Mouse satellite cell‐derived myoblasts were cultured in proliferation medium (A) or switched to differentiation medium for 2 days (B), fixed and immunolabelled for DEPDC1B (HPA072558) and β‐catenin. DEPDC1B was found in the nuclei of both proliferating satellite cell‐derived myoblasts and newly formed myotubes and unfused myocytes, but not at cell‐cell‐junctions. (C) Satellite cells were maintained in proliferation conditions or differentiation medium for 1 or 2 days and *Depdc1b* and *Pax7* expression measured by RT‐qPCR. *Depdc1b* was expressed in proliferating myoblasts, but then decreased during differentiation, mirroring expression of *Pax7*. (D) DEPDC1B was effectively knocked down via siRNA transfection (N1 and N2) compared to control siRNA (Si control), as assessed by RT‐qPCR. (E) Satellite cells transfected with control siRNA (Si control) or siRNA against DEPDC1B were maintained in proliferation conditions, and either pulsed for EdU and immunolabelled for MYOGENIN, or (G) immunolabelled for KI67 and β‐tubulin. (F) DEPDC1B knockdown reduced proliferation, but (H) promoted entry into differentiation. Data are mean ± SEM, where an asterisk denotes a significant difference (*P* < .05) between control and a test sample using a paired two‐tailed t test, with multiple fields examined from each of 3 mice. Scale bar represents 100 µm

Endogenous DEPDC1B protein was investigated in mouse primary satellite cells using a commercially available DEPDC1B antibody (HPA038255). DEPDC1B was clearly located in the nuclei of proliferating satellite cell‐derived myoblasts ex vivo (Figure 1A). After 2 days in differentiation medium, DEPDC1B remained in the nuclei of multinucleated myotubes and unfused cells (Figure 1B). Using isolated myofibres that retain satellite cells in their niche, we found that DEDPDC1B was not detectable in quiescent satellite cells, but was present at low levels in the nuclei of activated satellite cells after 24 h (Figure S1A,B). After 48 h ex vivo, DEPDC1B was robustly expressed in the nuclei of myogenin‐positive and myogenin‐negative satellite cells (Figure S1C). Immunolabelling for DEPDC1B in the nuclei of satellite cells was weaker after 72 h in culture (Figure S1D).

The expression profile of *Depdc1b* was assessed using RT‐qPCR. *Depdc1b* was expressed in proliferating satellite cell‐derived myoblasts, but expression decreased rapidly during myogenic differentiation into myocytes and myotubes assayed at day 1 or 2 of differentiation: an expression profile mirroring that of *Pax7* (Figure 1C).

DEPDC1B localization was also examined in mouse primary satellite cell‐derived myoblasts using a mouse DEPDC1B‐V5‐tagged construct. Satellite cells were transduced with a *Depdc1b‐V5*‐encoding retrovirus with an *IRES‐eGFP* (to identify transduced cells) and examined during proliferation as myoblasts (Figure S2A) or after 1 day of differentiation as myocytes and myotubes (Figure S2B) by co‐immunolabelling for V5 (DEPDC1B‐V5) and GFP. Exogenous DEPDC1B‐V5 was localized at the cell periphery and especially at points of cell‐cell contact (Figure S2A,B).

### 3.2 DEPDC1B regulates proliferation and prevents precocious differentiation in murine myoblasts

DEPDC1B function was investigated using siRNA‐mediated knockdown in murine satellite cells. *Depdc1B* expression was decreased by ~72% by two different Silencer selected pre‐designed siRNA targeting *Depdc1b* (N1 and N2) compared to transfection with control siRNA (Figure 1D). Western blot analysis (Figure S3A) confirmed that siRNA against *Depdc1B* also reduced DEPDC1B at the protein level (Figure S3B).

Satellite cells knocked down for DEPDC1B were pulsed with EdU for 2 hours in proliferation medium, fixed, EdU incorporation visualized, immunolabelled for MYOGENIN and counterstained with DAPI (Figure 1E). Knockdown of DEPDC1B decreased the rate of cell proliferation (EdU + ve) and the total number of cells (DAPI‐stained) (Figure 1F) compared to control siRNA. Reduction of cell proliferation following DEPDC1B knockdown was corroborated by a decreased proportion of KI67 + ve cells (Figure 1G,H).

Since proliferation was reduced, we focused more on the entry into myogenic differentiation, rather than fusion index, which is affected by cell number. DEPDC1B knockdown caused an increased entry into the myogenic differentiation programme, as shown by the higher proportion of myoblasts with MYOGENIN immunolabelling compared to siRNA control (Figure 1E,F).

We confirmed that siRNA‐mediated knockdown of DEPDC1B reduced the proliferation rate by also using a mix of 4 siRNAs targeting DEPDC1B (Qiagen). RT‐qPCR and Western blot analysis confirmed that these siRNAs also reduced *Depdc1b* mRNA (Figure S4A) and DEPDC1B at the protein level (Figure S3A) in murine primary myoblasts compared to control siRNA. DEPDC1B knockdown using these 4 separate siRNAs also significantly reduced the proliferation rate of satellite cell‐derived myoblasts, as shown by the decreased proportion that had incorporated EdU (Figure S4B,C).

### 3.3 DEPDC1B is required for proliferation in human myoblasts

**Figure 2:**
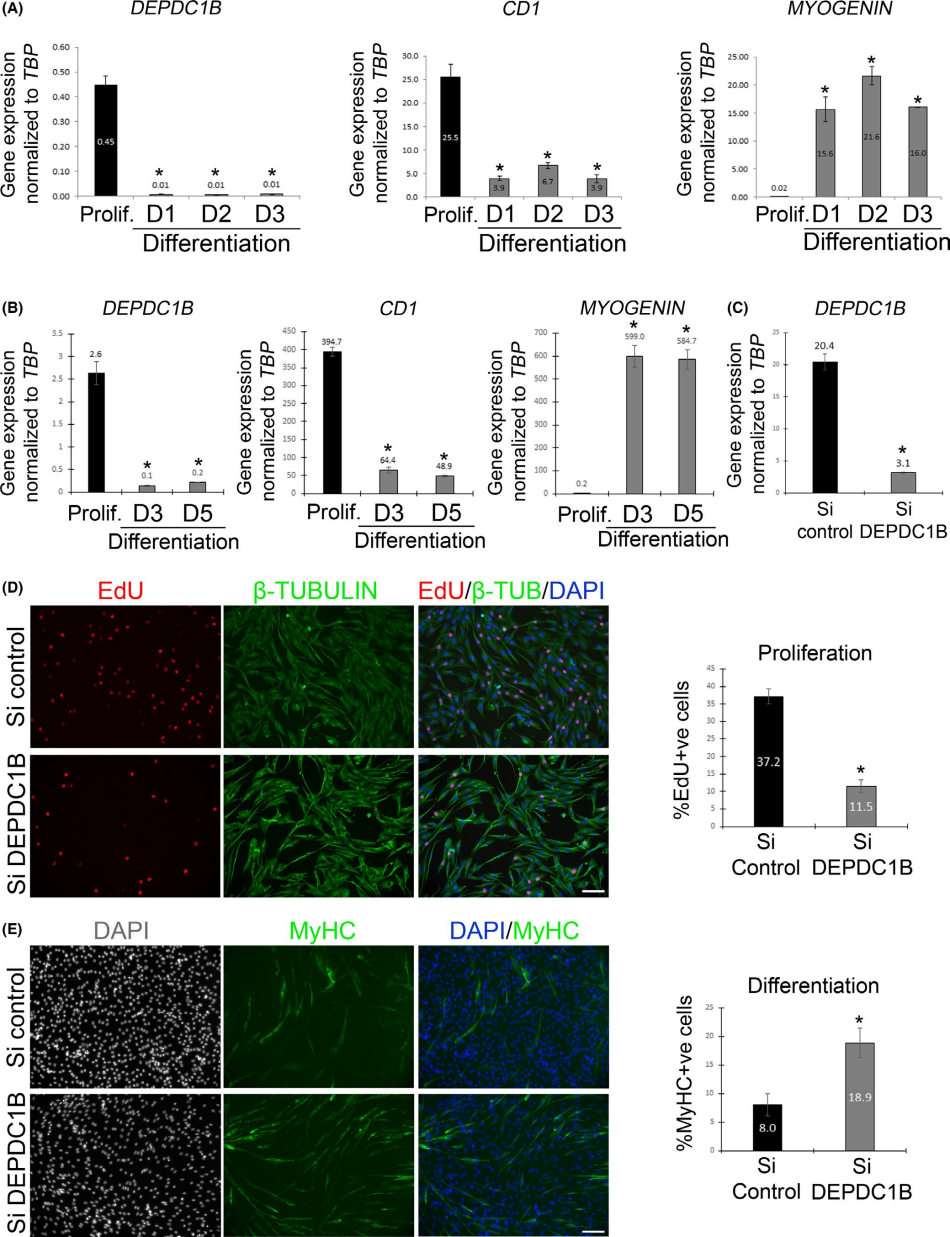
DEPDC1B controls proliferation and differentiation in human myoblasts. (A) cDNA from primary human myoblasts obtained from 3 individuals collected in proliferation, and at differentiation day 1, 2 or 3, was analysed for *DEPDC1B*, *CD1* and *MYOGENIN* expression by RT‐qPCR. *DEPDC1B* was expressed at a higher level in proliferating myoblasts than during differentiation, mirroring expression of *CD1*, but opposite to that of *MYOGENIN*. (B) Immortalized C25Cl48 human myoblasts were maintained in proliferation conditions or differentiation medium for 3 or 5 days; again, *DEPDC1B* was expressed at a higher level in proliferating myoblasts than differentiation, also emulating *CD1*, but contrary to the *MYOGENIN*, expression profile. (C) DEPDC1B knockdown efficiency via siRNA transfection was effective compared to control siRNA (Si control), as quantified using RT‐qPCR. (D) Control or DEPDC1B siRNA‐transfected C25Cl48 human myoblasts were maintained in proliferation medium, pulsed with EdU and immunolabelled for β‐tubulin. DEPDC1B knockdown decreased the proliferation rate. (E) DEPDC1B knockdown C25Cl48 myoblasts enter differentiation earlier, with an increased proportion of MyHC + ve cells after 2 days of differentiation. Data are mean ± SEM, where an asterisk denotes a significant difference (*P* < .05) between control and a test sample using either a paired (A) or an unpaired (B‐E) two‐tailed t test, with cDNA from 3 individuals or N = 3‐5 independently siRNA‐transfected wells analysed per condition. Scale bar represents 100 µm

Examining the expression dynamics of *DEPDC1B* during myogenesis in human primary myoblasts from three individuals, we found that *DEPDC1B* expression was significantly higher in proliferating myoblasts than in myocytes/myotubes at differentiation days 1, 2 or 5, mirroring cyclin *D1* (*CD1*) expression, and opposite to the expression profile of *MYOGENIN* (Figure 2A). A similar *DEPDC1B* expression profile was found in C25Cl48 immortalized human myoblasts in proliferation, compared to differentiation (Figure 2B). Examination of RNA‐Seq from control immortalized myoblasts through 8 time points during myogenic differentiation^46^ also showed that *DEPDC1B* expression fell during differentiation (Figure S5A). Interestingly, *DEPDC1B* expression was even lower during differentiation in a myoblast clone containing the D4Z4 contraction at chromosome 4q35 that causes facioscapulohumeral muscular dystrophy 1 (OMIM: 158900) (Figure S5A).

DEPDC1B knockdown was performed in C25Cl48 human myoblasts via transfection of a mix of four siRNAs (FlexiTube GeneSolution) targeting human *DEPDC1B* mRNA, with high knockdown efficiency compared to control siRNA, as confirmed by RT‐qPCR (Figure 2C). Following DEPDC1B knockdown, myoblasts were pulsed with EdU, which revealed a significantly reduced proliferation rate compared to control siRNA (Figure 2D). After 2 days in differentiation medium, immunolabelling for MyHC showed that DEPDC1B knockdown resulted in a higher proportion of differentiated myocytes compared to control siRNA (Figure 2E) suggesting that DEPDC1B normally operates to inhibit myogenic differentiation.

### 3.4 DEPDC1A does not affect proliferation in mouse or human myoblasts

**Figure 3:**
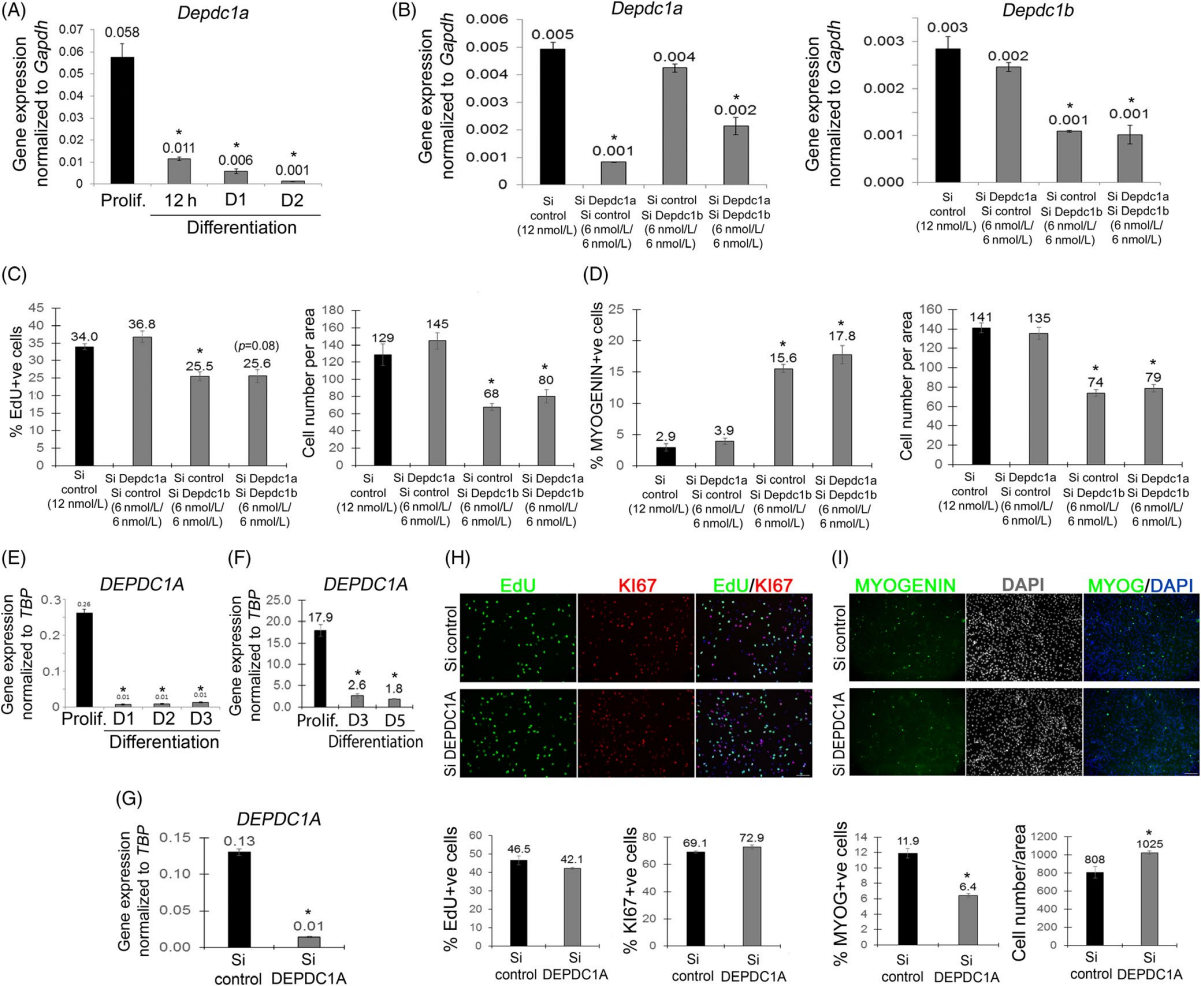
DEPDC1A does not regulate proliferation of myoblasts. (A) Murine satellite cells were maintained in proliferation medium or differentiation medium for 12 h, 1 or 2 days, and *Depdc1a* expression measured by RT‐qPCR. *Depdc1a* was expressed in proliferating satellite cell‐derived myoblasts, but rapidly down‐regulated during differentiation. (B) *Depdc1a* and *Depdc1b* were effectively knocked down individually or together via siRNA transfection compared to control siRNA (Si control), as shown by RT‐qPCR. (C, D) SiRNA‐transfected satellite cells were maintained in proliferation medium, pulsed with EdU and immunolabelled for MYOGENIN. (C) DEPDC1A knockdown did not affect cell proliferation or (D) differentiation, while DEPDC1A/DEPDC1B co‐knockdown did not have any additive effects. (E) cDNA from primary human myoblasts obtained from 3 individuals collected in proliferation, and at differentiation day 1, 2 or 3, or (F) immortalized human myoblasts (C25Cl48) in proliferation or at differentiation day 3 or 5 were analysed for *DEPDC1A* expression by RT‐qPCR. *DEPDC1A* was expressed at significantly higher levels in proliferating human myoblasts than during differentiation. (G) DEPDC1A knockdown via siRNA transfection was efficient, as shown by RT‐qPCR. (H) Control (Si control) and DEPDC1A siRNA‐mediated knocked down proliferating human myoblasts were pulsed with EdU and immunolabelled for Ki67. DEPDC1A knockdown did not affect myoblast proliferation. (I) SiRNA‐transfected human myoblasts were maintained in differentiation medium for 1 day, fixed and immunolabelled for MYOGENIN. DEPDC1A knockdown inhibited the entry into myogenic differentiation, and increased total cells. Data are mean ± SEM, where an asterisk denotes a significant difference (*P* < .05) between control and a test sample using a paired (A‐E) or unpaired (F‐I) two‐tailed t test, with multiple fields examined from either N = 3 mice, N = 3 individuals or 3 or more independent wells. Scale bar represents 100 µm

DEPDC1A has functional redundancy with DEPDC1B in control of cell cycle progression.^1^ DEPDC1A had a similar expression pattern to *DEPDC1B* in mouse satellite cell‐derived myoblasts (Figure 3A), human primary myoblasts (Figure 3E) and human C25Cl48 myoblasts (Figure 3F)*,* with *DEPDC1A* expressed at significantly higher levels during proliferation, when compared to differentiation.

siRNA‐mediated knockdown efficiency for *DEPDC1A* was validated by RT‐qPCR in both mouse (Figure 3B) and human C25Cl48 (Figure 3G) myoblasts. In mouse, DEPDC1A knockdown did not reduce the proliferation rate as tested by measuring EdU incorporation (Figure 3C) or number of myoblasts (Figure 3C,D), unlike knockdown of DEPCD1B. DEPDC1A knockdown did not affect differentiation either, as assessed by MYOGENIN immunolabelling (Figure 3D). Moreover, co‐knockdown of both DEPDC1A and DEPDC1B together did not have an additive effect on proliferation rate or differentiation, compared to DEPDC1B knockdown alone (Figure 3C,D). Similarly, DEPDC1A knockdown in human C25Cl48 myoblasts did not affect the proliferation rate as assessed by the proportion of EdU or Ki67 + ve cells (Figure 3H). DEPDC1A knockdown in human myoblasts though did decrease the proportion of MYOGENIN + ve cells, with more myoblasts (data not shown) and myocytes per unit area (Figure 3I).

### 3.5 DEPDC1B affects key regulators of the cell cycle

**Figure 4:**
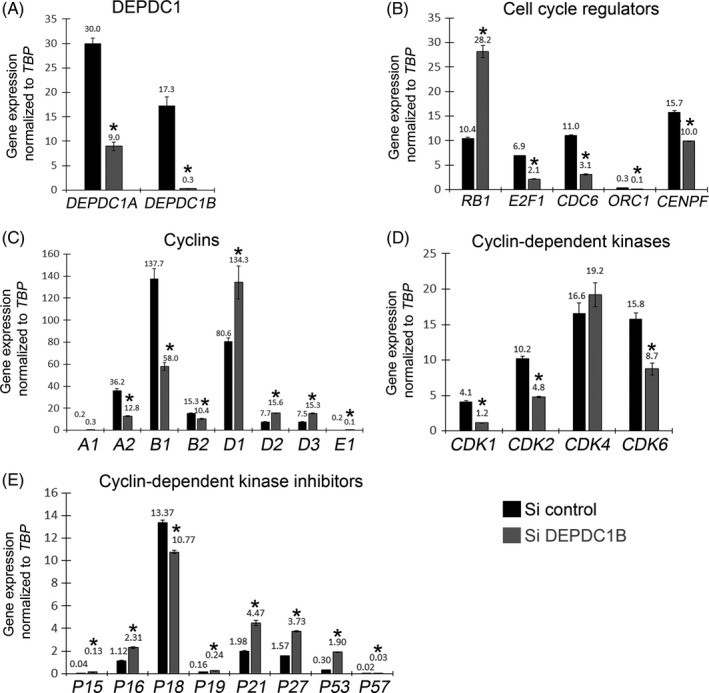
DEPDC1B knockdown myoblasts exhibit deregulation of cell cycle genes. Human C25Cl48 myoblasts were transfected with a siRNA control (Si control) or a siRNA against DEPDC1B (Si DEPDC1B). Forty‐eight hours post‐transfection in proliferation medium, RNA was extracted and expression of cell cycle genes analysed by RT‐qPCR. (A) DEPDC1B knockdown was confirmed and compared to control myoblasts. (B‐E) Cell cycle inhibitors were up‐regulated (*RB1, P15, P16, P19, P21, P27, P53, P57)*, while genes required for normal cell cycle progression were down‐regulated (*E2F1; Cyclins A2, B1, B2, E1; CDk1, CDK2, CDK6)*. Data are mean ± SEM, where an asterisk denotes a significant difference (*P* < .05) between control and a test sample using an unpaired two‐tailed t test, with N = 3 independently siRNA‐transfected wells analysed per condition

To characterize the effects of knocking down DEPDC1B in proliferating human C25Cl48 myoblasts, we analysed expression of many key genes involved in regulation of cell cycle progression, including cyclins, cyclin‐dependent kinases and cyclin‐dependent kinase inhibitors (Figure 4). Proliferating human myoblasts were transfected with siRNA control or siRNA against DEPDC1B and maintained in proliferation conditions for 48 h. *DEPDC1B* knockdown was confirmed, although *DEPDC1A* was also significantly affected (Figure 4A). Consistent with reduced proliferation, we detected an increase in *RB1* expression and a decrease in *E2F1*, *CDC6*, *ORC1* and *CENPF* (Figure 4B). Cyclins *A2*, *B1*, *B2* and *E1* were also decreased compared to control siRNA, while cyclins *D1*, *D2* and *D3* increased (Figure 4C). Cyclin‐dependent kinases *CDK1*, *CDK2* and *CDK6* were decreased (Figure 4D), while the cyclin‐dependent kinase inhibitors *P15*, *P16*, *P19*, *P21*, *P27*, *P53* and *P57* were all significantly increased compared to control siRNA (Figure 4E).

### 3.6 PITX2 does not control DEPDC1B in human myoblasts

*DEPDC1B* is repressed by PITX2, with PITX2 knockdown increasing DEPDC1B at the protein level in mouse C2C12 myoblasts.^6^ To test whether this regulatory mechanism is conserved in man, we first analysed *PITX2* expression. *PITX2* expression in immortalized human C25Cl48 myoblasts increased during differentiation (Figure S6A). As a repressor of DEPDC1B, such an increase in *PITX2* could explain the reduction of *DEPDC1B* in both human myocytes and myotubes (Figure 2A,B). To test this hypothesis, human immortalized C25Cl48 myoblasts were transduced with retrovirus encoding *PITX2C*, and overexpression was validated by RT‐qPCR (Figure S6B). However, retroviral‐mediated overexpression of PITX2C did not repress *DEPDC1B* expression in proliferating human myoblasts (Figure S6B), and neither did overexpression of PITX1 or PITX3 (data not shown). We also performed the complementary experiment of knocking down *PITX2* expression via siRNA and analysing *DEPDC1B* expression after 2 days of differentiation (Figure S6C). Knockdown efficiency of *PITX2* siRNA was validated (although it also affected *Pitx1* and *Pitx3*), but no increased *DEPDC1B* expression was measured (Figure S6C).

### 3.7 DEPDC1B functions independently of WNT/β‐catenin signalling in myogenic cells

In cancer cells, DEPDC1B affects WNT/β‐catenin signalling^33^ a pathway also involved in satellite cell regulation.^47^, ^48^ Therefore, we performed a double DEPDC1B/β‐catenin siRNA‐mediated knockdown rescue experiment in murine satellite cells, to test whether the reduced proliferation/precocious differentiation observed by knocking down DEPDC1B alone could be attributed to activation of β‐catenin. Efficient knockdown of DEPDC1B and/or β‐catenin compared to control siRNA was confirmed by RT‐qPCR (Figure S7A). DEPDC1B knockdown again reduced proliferation (KI67 + ve myoblasts) and enhanced differentiation (MYOGENIN + ve myoblasts), but knockdown of β‐catenin did not affect either proliferation or differentiation compared to control siRNA. Knockdown of both DEPDC1B and β‐catenin did not rescue the effects on proliferation or differentiation compared to DEPDC1B knockdown alone (Figure S7B).

### 3.8 Synergistic suppression of proliferation but enhancement of differentiation by DEPDC1B and RHOA co‐knockdown in human myoblasts

DEPDC1B knockdown causes a proliferation defect in human HeLa cells by an increase in RHOA activation and impairment of the de‐adhesion process.^1^ PTPRF is required for RHOA activation, and DEPDC1B inactivates RHOA by competing for binding of PTPRF. Knockdown of RHOA or PTPRF was able to rescue the proliferation defect of DEPDC1B knockdown HeLa cells.^1^

**Figure 5:**
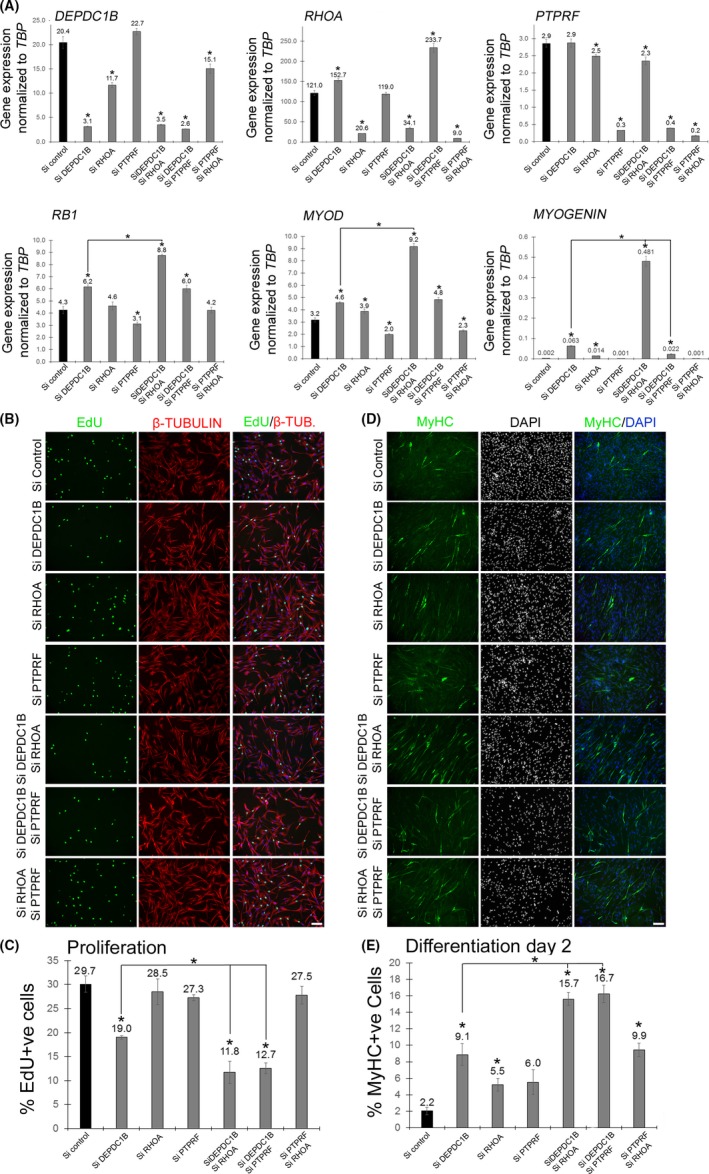
DEPDC1B and RHOA synergize in suppressing proliferation but enhancing differentiation in human myoblasts. (A) Immortalized human C25Cl48 myoblasts were transfected with control siRNA (Si control) or siRNA against DEPDC1B, RHOA, PTPRF, DEPDC1B/RHO, DEPDC1B/PTPRF or PTPRF/RHOA. Transfected cells were maintained in proliferation medium, mRNA was extracted, and expression of *DEPDC1B*, *RHOA*, *PTPRF*, *RB1*, *MYOD* and *MYOGENIN* was analysed by RT‐qPCR. DEPDC1B knockdown cells had increased *RB1*, *MYOD* and *MYOGENIN* expression. RHOA knockdown caused an increase in *MYOD* and *MYOGENIN*. However, DEPDC1B/RHOA double knockdown caused a strong additive increase in *RB1*, *MYOD* and *MYOGENIN* expression. (B) SiRNA‐transfected human C25Cl48 myoblasts were maintained in proliferation, pulsed with EdU, fixed and immunolabelled for β‐TUBULIN. (C) A reduction of the proliferation rate was observed after DEPDC1B knockdown, but not with RHOA knockdown. Double DEPDC1B/RHOA or DEPDC1B/PTPRF knockdown had a synergistic effect in suppressing proliferation. (D) SiRNA‐transfected human C25Cl48 myoblasts were maintained in differentiation medium for 2 days, fixed and immunolabelled for MyHC. Differentiation was induced in DEPDC1B knockdown and RHOA knockdown myoblasts, with an additive effect in double DEPDC1B/RHOA or DEPDC1B/PTPRF knockdown cells. Data are mean ± SEM, where an asterisk denotes a significant difference (*P* < .05) between control and a test sample, or as indicated with a bar, using an unpaired two‐tailed t test, with 3 separately siRNA‐transfected wells (RT‐qPCR) or multiple fields analysed from 3 separate wells (immunolabelling) per condition. Scale bar equals 100 µm

To test this mechanism in human C25Cl48 myoblasts, DEPDC1B, RHOA and PTPRF were knocked down via siRNA transfection individually, or in combination, for 48 hours (Figure 5A). Knockdown of RHOA reduced *DEPDC1B* expression, while DEPDC1B knockdown or DEPDC1B/PTPRF knockdown increased *RHOA* expression compared to control siRNA (Figure 5A). DEPDC1B knockdown induced an increase in *RB1*, *MYOD* and *MYOGENIN* compared to control siRNA*,* which further supports the observed phenotype of suppressed proliferation and augmented differentiation. RHOA knockdown increased *MYOD* and *MYOGENIN* compared to control siRNA. PTPRF knockdown induced a moderate reduction of *RB1* and *MYOD*. Unexpectedly, co‐knockdown of DEPDC1B and RHOA caused a larger increase in *RB1*, *MYOD* and *MYOGENIN* expression than DEPDC1B knockdown alone, so enhancing, rather than reversing, the effect of DEPDC1B knockdown in promoting differentiation (Figure 5A).

siRNA‐transfected human proliferating C25Cl48 myoblasts were also pulsed with EdU and immunolabelled for β‐tubulin (Figure 5B) or for MyHC and DAPI after 2 days of differentiation (Figure 5D). As expected from Figure 2, DEPDC1B knockdown decreased the proportion of human myoblasts that had incorporated EdU compared to control siRNA. In contrast, RHOA or PTPRF knockdown did not affect the proliferation rate. However, DEPDC1B/RHOA or DEPDC1B/PTPRF co‐knockdown caused a further reduction in the proliferation rate compared with DEPDC1B knockdown alone (Figure 5C).

Again as expected from Figure 2, DEPDC1B knockdown increased the proportion of MyHC + ve myocytes compared to control siRNA. RHOA knockdown also increased differentiation, while PTPRF knockdown did not have any measurable effect. Simultaneous DEPDC1B/RHOA knockdown caused a significantly more substantial increase in differentiation of human myoblasts compared to knockdown of either DEPDC1B or RHOA alone, as did double DEPDC1B/PTPRF compared to knockdown of either DEPDC1B or PTRF alone (Figure 5E).

### 3.9 DEPDC1B knockdown increases myogenic differentiation in Rhabdomyosarcoma cells

DEPDC1B is overexpressed in many cancers, where it promotes their proliferation, migration and invasion. We therefore examined *DEPDC1B* expression in rhabdomyosarcoma (RMS),^38^ the most common soft tissue sarcoma in children and adolescents. There are two major subtypes of RMS, embryonal (ERMS) and alveolar (ARMS). We examined *DEPDC1B* expression in rhabdomyosarcoma tumours using published RNA‐Seq data sets (http://www.ncbi.nlm.nih.gov/geo/query/acc.cgi?acc=GSE28511), profiling ARMS and ERMS alongside control skeletal muscle and tumour adjacent skeletal muscle. This analysis revealed that *DEPDC1B* was expressed at a higher level in rhabdomyosarcoma tumours compared to the skeletal muscle biopsies (Figure S5B).

**Figure 6:**
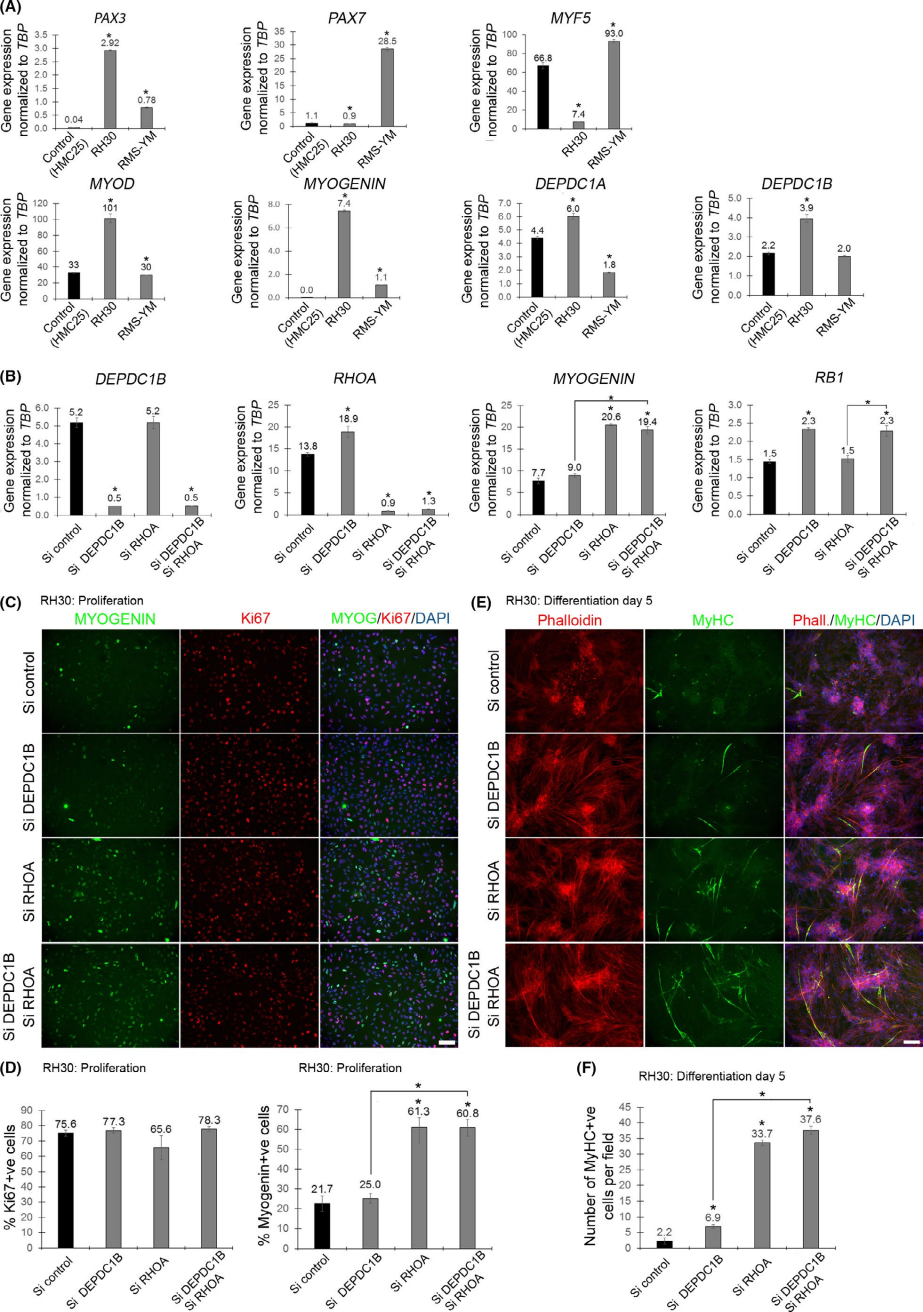
DEPDC1B or RHOA knockdown enhance myogenic differentiation in Rhabdomyosarcoma cells. (A) Control human C25Cl48 myoblasts (HCM25), Rh30 and RMS‐YM cells were maintained in proliferation medium, mRNA was extracted, and expression of *PAX3, PAX7, MYF5, MYOD, MYOGENIN, DEPDC1A* and *DEPDC1B* was analysed by RT‐qPCR. *DEPDC1A* or *DEPDC1B* were expressed at higher levels in Rh30 cells compared to control human myoblasts. (B) Rh30 cells were transfected with either control siRNA (Si control) or siRNA against DEPDC1B and/or RHOA, mRNA was extracted, and expression of *DEPDC1B*, *RHOA*, *MYOGENIN* and *RB1* was analysed by RT‐qPCR. *DEPDC1B* and/or *RHOA* knockdown were validated, and *MYOGENIN* was only induced in RHOA knockdown cells and *RB1* only in DEPDC1B knockdown cells. No additive effects were observed with DEPDC1B/RHOA double knockdown. (C) SiRNA‐transfected Rh30 cells were maintained in proliferation, fixed and immunolabelled for KI67 and MYOGENIN. (D) No reduction in proliferating cells was observed with DEPDC1B knockdown, RHOA knockdown or double DEPDC1B/RHOA knockdown. However, the number of MYOGENIN‐positive cells was increased only by RHOA knockdown. (E) SiRNA‐transfected Rh30 cells were maintained in differentiation medium for 5 days, fixed and stained with Phalloidin and immunolabelled for MyHC. (F) Cell differentiation was slightly but significantly enhanced by DEPDC1B knockdown, but more robustly increased by RHOA knockdown, with a trend (*P* = .06) towards an additive effect with double DEPDC1B/RHOA knockdown compared to RHOA knockdown alone. Data are mean ± SEM, where an asterisk denotes a significant difference (*P* < .05) between control and a test sample, or as indicated by a bar, using an unpaired two‐tailed t test, with 3 separately siRNA‐transfected wells (RT‐qPCR) or multiple fields per 3 wells (immunolabelling) per condition. Scale bar equals 100 µm

We then measured *DEPDC1B* and *DEPDC1A* expression in two different RMS cells lines in proliferating conditions, Rh30 (ARMS) and RMS‐YM (ERMS), and compared levels to those in control human C25Cl48 myoblasts. *DEPDC1A* and *DEPDC1B* were expressed at a higher level in Rh30 cells, compared to C25CL48 myoblasts (Figure 6A). Rh30 cells had higher expression of *PAX3*, *MYOD* and *MYOGENIN*, but less *PAX7* and *MYF5,* while RMS‐YM had higher expression of *PAX3, PAX7, MYF5* and *MYOGENIN,* when compared to control C25CL48 myoblasts (Figure 6A).

DEPDC1B and RHOA co‐knockdown synergistically inhibits proliferation but promotes differentiation in control human C25CL48 myoblasts (Figure 5). To test whether DEPDC1B and RHOA had similar effects in ARMS, Rh30 were transfected with control siRNA and siRNA against DEPDC1B and/or RHOA for 48 h in proliferative conditions (Figure 6B). DEPDC1B knockdown caused an increase in *RHOA* expression in Rh30 cells*. RB1* expression was increased in Rh30 cells knocked down for DEPDC1B, but *MYOGENIN* expression was unchanged compared to control siRNA. Increased *MYOGENIN* expression was only observed in RHOA knocked down Rh30 cells. Rh30 cells knocked down for both DEPD1B and RHOA exhibited no synergetic induction on *RB1* or *MYOGENIN* expression (Figure 6B).

Next, siRNA‐transfected Rh30 cells were maintained in proliferation conditions for 2 days and immunolabelled for KI67 and MYOGENIN (Figure 6C) or cultured in differentiation conditions for 5 days, and then immunolabelled for MyHC and stained with phalloidin (Figure 6E). In proliferating conditions, DEPDC1B and/or RHOA knockdown did not affect Rh30 proliferation. However, the proportion of MYOGENIN + ve cells was increased in RHOA knockdown cells compared to control siRNA, but unaffected by DEPDC1B knockdown (Figure 6D). After 5 days in differentiation conditions, DEPDC1B knockdown induced a slight increase in the number of differentiated MyHC + ve cells per unit area compared to control siRNA. RHOA knockdown had a more robust effect in promoting MyHC expression in Rh30 cells. DEPDC1B/RHOA co‐knockdown trended towards further enhancing the number of differentiated MyHC + ve cells per unit area (*P* = .06), compared to RHOA knockdown alone (Figure 6F). As a proportion of total Rh30 cells though, those expressing MyHC were a minor component.

## 4 DISCUSSION

*DEPDC1B* is a cell cycle–regulated gene that is highly expressed during the G2/M phase of the cell cycle.^1^ DEPDC1B plays a role in cell cycle progression and is overexpressed in many cancers.^34^ Here, we analysed DEPDC1B function during skeletal myogenesis.

*DEPDC1B* is expressed in proliferating mouse and human myoblasts, but levels rapidly decrease during differentiation, in agreement with DEPDC1B being almost undetectable in human skeletal muscle.^5^ At the protein level, DEPDC1B was present in the nucleus in both proliferating murine myoblasts and differentiated multinucleated myotubes, indicating that while *DEPDC1B* expression is actively repressed during differentiation, the protein could remain functional in newly formed myotubes. The DEPDC1B antibody (HPA072558) recognizes a band of the correct molecular weight for DEPDC1B at approximately 61 kDa on Western blots of proteins from mouse myoblasts (Figure S3) and is located in the nucleus in various cancer cell lines.^37^ However, DEPDC1B is often reported to also be located at the cell membrane.^1^, ^3^, ^6^ Interestingly, when we overexpressed a V5‐tagged version of DEPDC1B in mouse myoblasts, we found clear membrane localization (Figure S2). In human C25Cl48 myoblasts though, we did not observe nuclear localization with the HPA072558 DEPDC1B antibody, although this antibody did not recognize a band at ~ 61 kDa on Western blots, instead recognizing a band around 42 kDa (unpublished data).

To evaluate DEPDC1B function, we used a knockdown approach via siRNA transfection. DEPDC1B knockdown in mouse and human myoblasts caused a similar phenotype, characterized by a robust reduction in their proliferation and deregulation of many cell cycle genes. During the G1 phase, RB1 inactivates E2F1 to block G1/S transition. DEPDC1B knockdown myoblasts had higher expression of *RB1* and a reduction of *E2F1* that could potentially cause a block in G1. DEPDC1B knockdown myoblasts also exhibited a reduction of markers that are normally increased during S phase (*cyclin A*, *CDK1* and *2)* or G2 phase *(cyclin B1* and *B2)*. Finally, DEPDC1B knockdown cells had increased expression of the CDK inhibitors *P21*, *P27*, *P53* and *P57* that inhibit cell cycle progression at different stages. Similar observations have been made in other cells types, for example DEPDC1B knockdown also inhibits proliferation in HeLa^1^ and human malignant melanoma cells.^34^ In parallel with inhibiting proliferation, DEPDC1B knockdown myoblasts also undergo precocious myogenic differentiation.

DEPDC1A has a similar structure to DEPDC1B and is also regulated during the cell cycle, with DEPDC1A knockdown in HeLa cells causing a similar phenotype to DEPDC1B knockdown, with simultaneous depletion of both DEPDC1A and DEPDC1B having additive effects.^1^ We found that *DEPDC1A* has a similar expression profile to *DEPDC1B* in mouse and man, with expression clearly falling in differentiation compared to levels during cell cycle. However, DEPDC1A knockdown had no effect on murine myoblast proliferation or entry into differentiation and did not act synergistically with DEPDC1B. It is of note that in human myoblasts, while DEPDC1A knockdown did not affect EdU incorporation or Ki67 expression, it did lead to an increase in cell number per unit area and fewer cells containing MYOGENIN. Thus, proliferation in myoblasts is supported by DEPDC1B, with DEPDC1A not appearing to have a major role.

To investigate the transcriptional regulation of DEPDC1B, we examined PITX2, which has been reported to be a repressor of DEPDC1B in murine immortalized C2 myoblasts.^6^ To test whether this regulatory mechanism was conserved in human myoblasts, we overexpressed PITX2C or knocked down PITX2, but neither procedure affected *DEPDC1B* expression. Thus, PITX2 does not appear to have a role in direct transcriptional regulation of *DEPDC1B* in human myoblasts. Further investigations will be required to identify protein/DNA‐binding elements for other factors involved in myogenesis located in *DEPDC1B* regulatory regions.

To better understand how DEPDC1B regulates myoblast proliferation, we investigated pathways described in other systems. DEPDC1B is overexpressed in non‐small‐cell lung carcinoma, where it can enhance both cell migration and invasion, an effect partially due to activation of canonical WNT signalling through β‐catenin.^33^ In mouse, while Wnt/β‐catenin signalling is transiently active in myoblasts during muscle regeneration in adult, genetic inactivation of β‐catenin in satellite cells does not affect regeneration.^49^ Interestingly though, constitutive activation of β‐catenin in satellite cells extends the myoblast phase of regeneration to ultimately generate smaller fibres and increase muscle fibrosis.^49^ We previously reported that inhibiting Axin1/2 in satellite cells increases signalling via β‐catenin to cause inhibited proliferation and precocious entry into differentiation.^47^ Thus, DEPDC1B knockdown caused a phenotype similar to activation of β‐catenin.^47^ We therefore tried to rescue DEPDC1B knockdown by also down‐regulating β‐catenin, but DEPDC1B/β‐catenin double knockdown did not rescue proliferation or precocious differentiation. Therefore, DEPDC1B function is not directly associated with β‐catenin levels in skeletal myogenesis.

DEPDC1B plays a role in control of cell cycle progression in some cells via a repressive effect on the small RHO GTPase RHOA.^1^ DEPDC1B inactivates RHOA by competing for binding of PTPRF. In the absence of DEPDC1B, RHOA activation is maintained and cell cycle progression inhibited. To determine whether a potential overactivation of RHOA was responsible for the proliferation defect observed in DEPDC1B knocked down human myoblasts, we reduced levels of DEPDC1B and/or RHOA via siRNA. Contrary to HeLa cells, RHOA reduction in DEPDC1B knockdown human myoblasts was unable to rescue proliferation. Surprisingly though, simultaneous down‐regulation of DEPDC1B and RHOA actually acted synergistically to further reduce cell proliferation and induce differentiation, with a synergistic increase in *RB1*, *MYOD* and *MYOGENIN* expression. DEPDC1B plays a key role in the maintenance of cell proliferation, and complementarily, RHOA inhibits myogenic differentiation. Knocking down both genes therefore allows a synergetic induction of differentiation, with withdrawal from cell cycle being a prerequisite for myogenic differentiation.

RHOA promotes myogenic differentiation but needs to be down‐regulated to then allow fusion.^8^, ^11^, ^13^, ^15^, ^16^, ^17^ In human myoblasts, we found that RHOA knockdown does not affect proliferation, but causes precocious entry to differentiation, suggesting an inhibitory effect of RHOA on induction of myogenic differentiation. The active form of RHOA is high in proliferation and at the later stages of differentiation, but not at the initiation of differentiation,^50^ supporting a potential inhibitory role of RHOA on differentiation. However, in proliferating murine C2C12 myoblasts, overexpression of a dominant‐negative RHOA (N19‐RhoA)^11^ or RHO‐specific inhibitor (tat‐C3)^17^ inhibits myogenic differentiation. These contradictory results could be due to cell line differences (immortalized murine C2C12 versus immortalized human myoblasts) or in the strategy to either inactivate RHOA or reduce its levels via siRNA. A specific level and temporal activation of RHOA seem to be important to allow coordinated myogenic progression.^17^

DEPDC1B is overexpressed in many cancers,^3^, ^35^, ^36^ and we found higher expression of *DEPDC1B* in human rhabdomyosarcoma compared to skeletal muscle, and in Rh30 (ARMS) cells compared to human C25Cl48 myoblasts. Knockdown of DEPDC1B in Rh30 cells increased expression of *RB1*, but not *MYOGENIN*. However, DEPDC1B knockdown did not otherwise affect proliferation (proportion of cells with KI67) or entry into myogenic differentiation (proportion of cells with MYOGENIN). However, after 5 days in differentiation conditions, the number of cells with the sarcomeric protein MyHC was moderately increased by DEPDC1B knockdown. RHOA knockdown alone in Rh30 triggered an increase in *MYOGENIN* expression and a higher number of cells with MYOGENIN and MyHC. This still represented only a small proportion of total cells, but MyHC containing Rh30 cells had presumably exited cell cycle. While we found that siRNA against RHOA did not affect Rh30 proliferation, dominant‐negative RHOAN19 has been reported to reduce rhabdosphere formation in multiple RMS cell lines.^51^ A constitutively active RHOA (RHOAV14), however, blocks myogenic differentiation (*MCad, MyoD, MyoG* expression) to retain ERMS cells in a more undifferentiated and self‐renewing state.^51^ Thus, in human myoblasts and Rh30 cells, RHOA knockdown promotes myogenic differentiation, supporting a role for RHOA as an inhibitor of precocious differentiation.

There are other pathways that DEPDC1B may also interact with. RAC1 is regulated by DEPDC1B in some cell lines^5^, ^6^ and in muscle cells, and RAC1 inhibits myogenic differentiation and induces loss of cell contact inhibition. Moreover, RAC1 is activated in rhabdomyosarcoma cell lines, and overexpression of dominant‐negative forms inhibits cell proliferation of RMS.^22^ Therefore, a potential interaction of DEPDC1B and RAC1 could occur in muscle. DEPDC1B overexpression also increases phosphorylation of ERK,^3^, ^5^ and the MEK/ERK pathway controls myoblast proliferation, while MEK inhibition in RAS‐mutated rhabdomyosarcoma can induce myogenic differentiation. Finally, N‐cadherin acts to maintain satellite cells in quiescence.^52^ N‐cadherin‐dependent intercellular adhesion also has a major role in cell cycle exit and in induction of the muscle differentiation programme via a positive regulation of RHOA and a negative regulation of RAC1, CDC42 and JNK activities.^53^ N‐cadherin knockdown in C2 myoblasts causes an increase in cell proliferation and a defect of differentiation characterized by a decrease in *p21*, *p27* and *MYOGENIN*. DEPDC1B knockdown has an opposite phenotype, with a defect of proliferation and an induction of differentiation with an increase in *p21*, *p27* and *Myogenin*. This could suggest a potential repressive effect of DEPDC1B on N‐cadherin.

In summary, *DEPDC1B* is expressed in proliferating myoblasts where it is localized to the nucleus in mouse. DEPDC1B functions in mouse and man to maintain myoblast proliferation and inhibit precocious entry into myogenic differentiation during adult skeletal myogenesis.

## CONFLICT OF INTEREST

The authors declare no potential conflicts of interest.

## Data Availability

The data that support the findings of this study are available from the corresponding author upon reasonable request.
