# Supplementary figures and images for: DEPDC1B is a key regulator of myoblast proliferation in mouse and man
Source: Cell Prolif. 2019 Dec 11;53(1):e12717. doi: 10.1111/cpr.12717 (PMC6985657; doi:10.1111/cpr.12717)

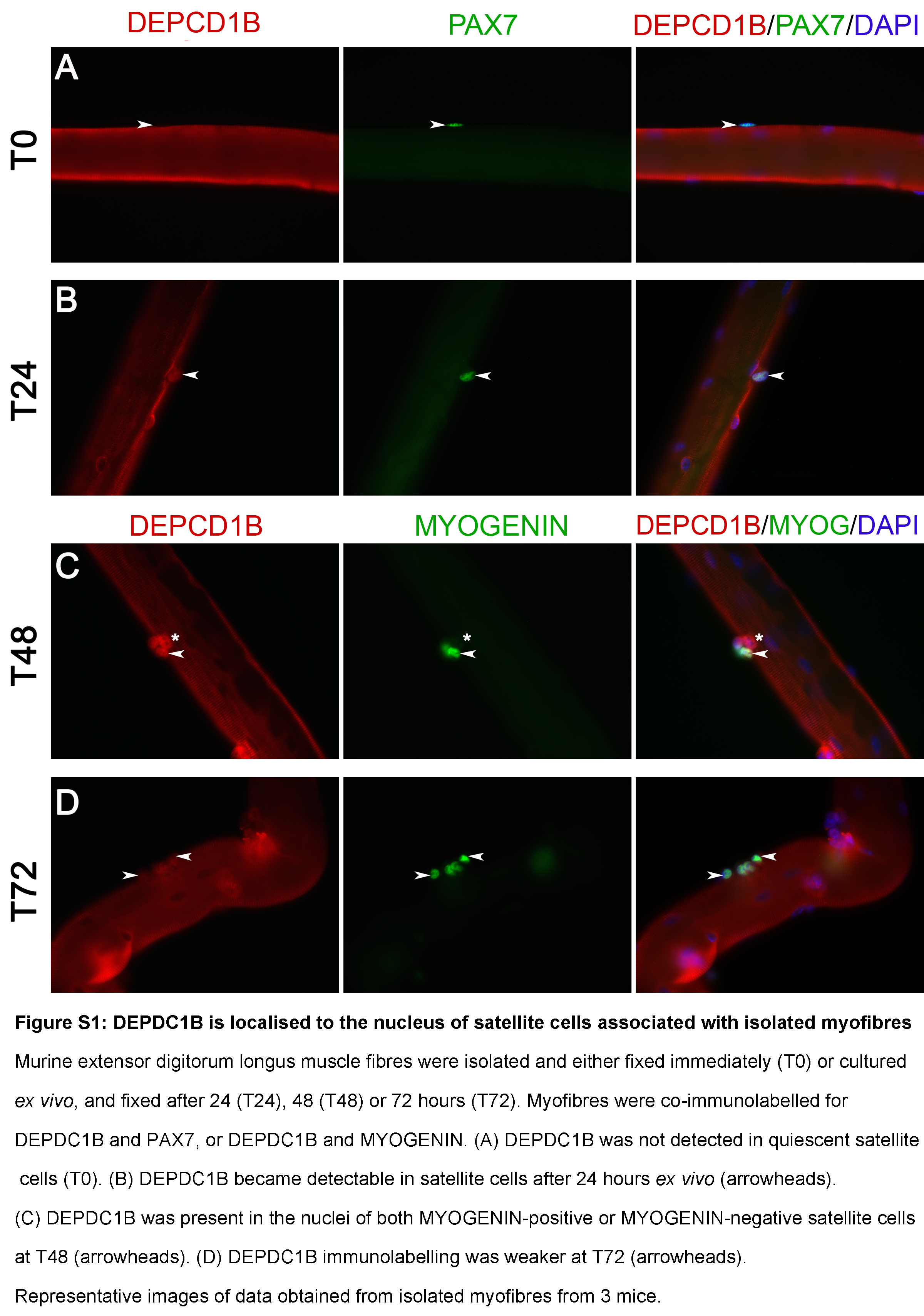

Supplement: Supplementary file 1 [file CPR-53-e12717-s001.jpg]

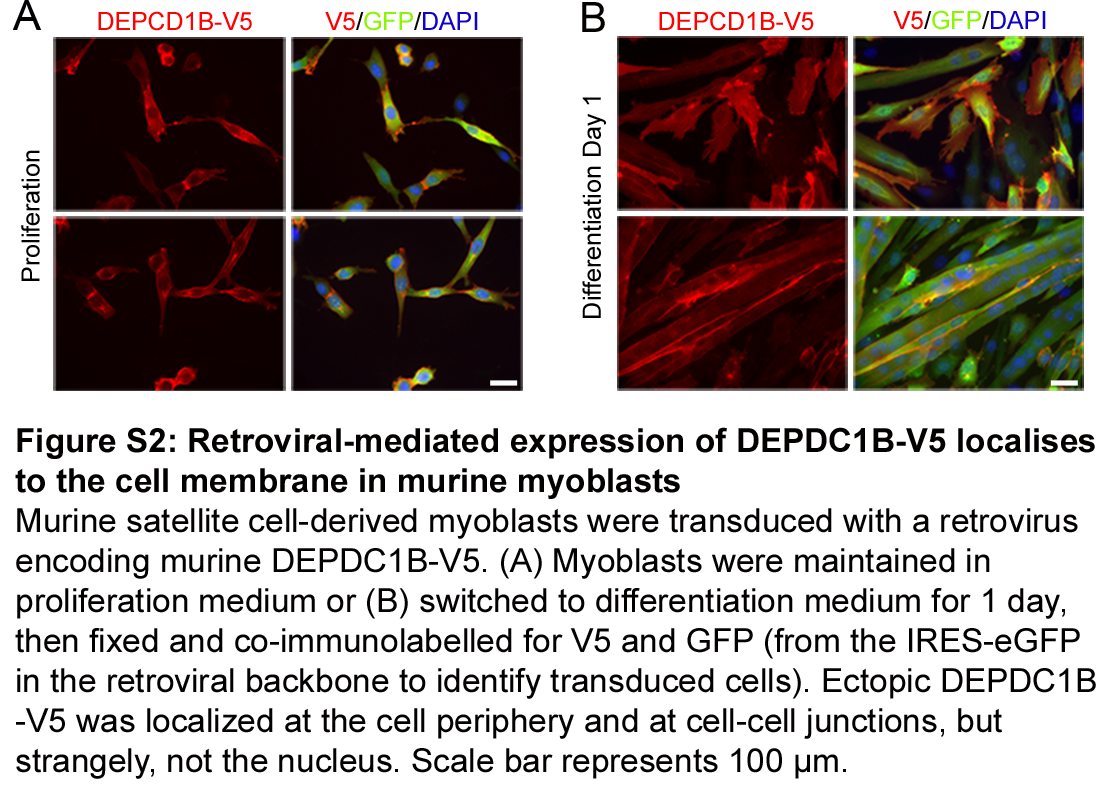

Supplement: Supplementary file 2 [file CPR-53-e12717-s002.tif]

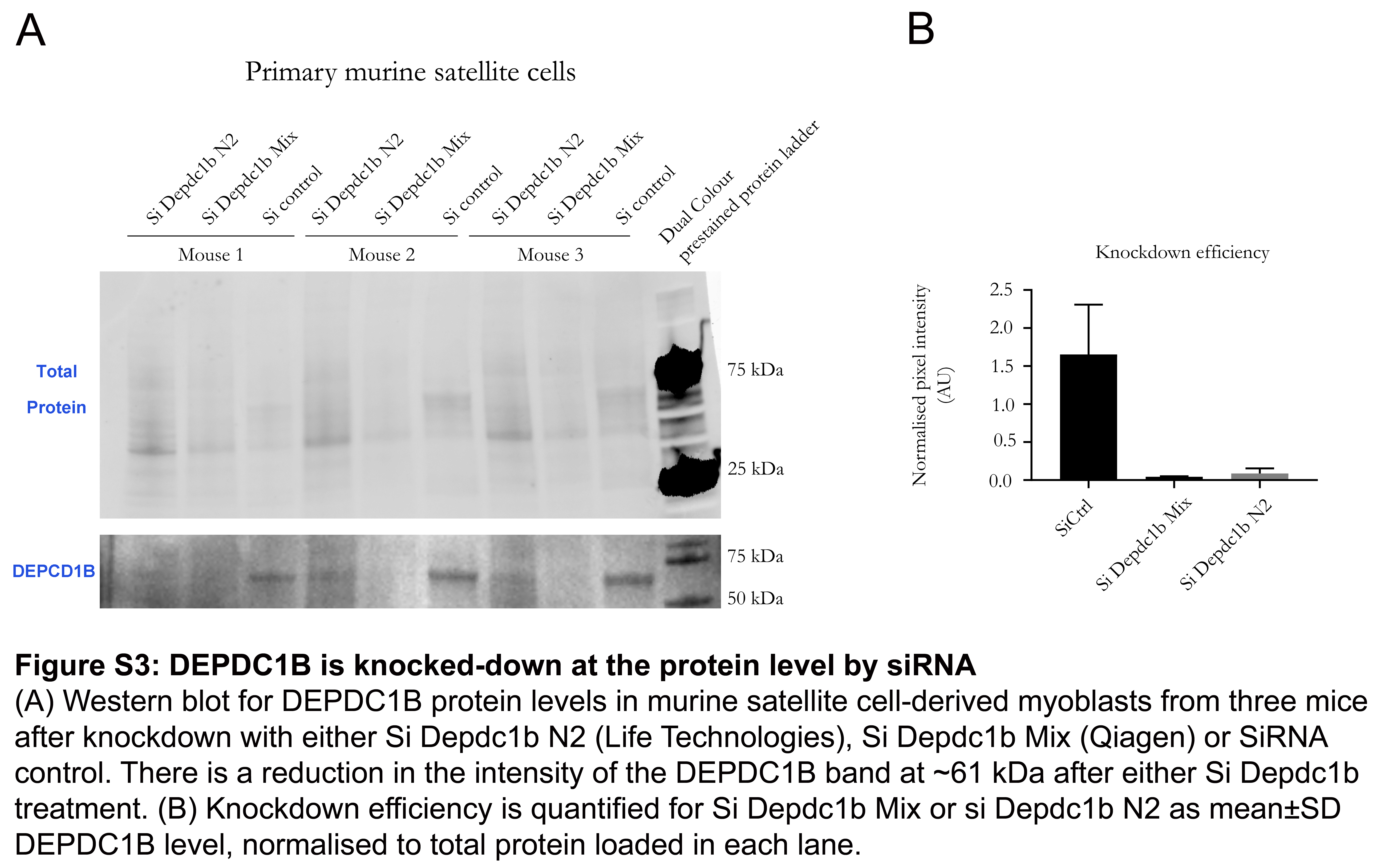

Supplement: Supplementary file 3 [file CPR-53-e12717-s003.jpg]

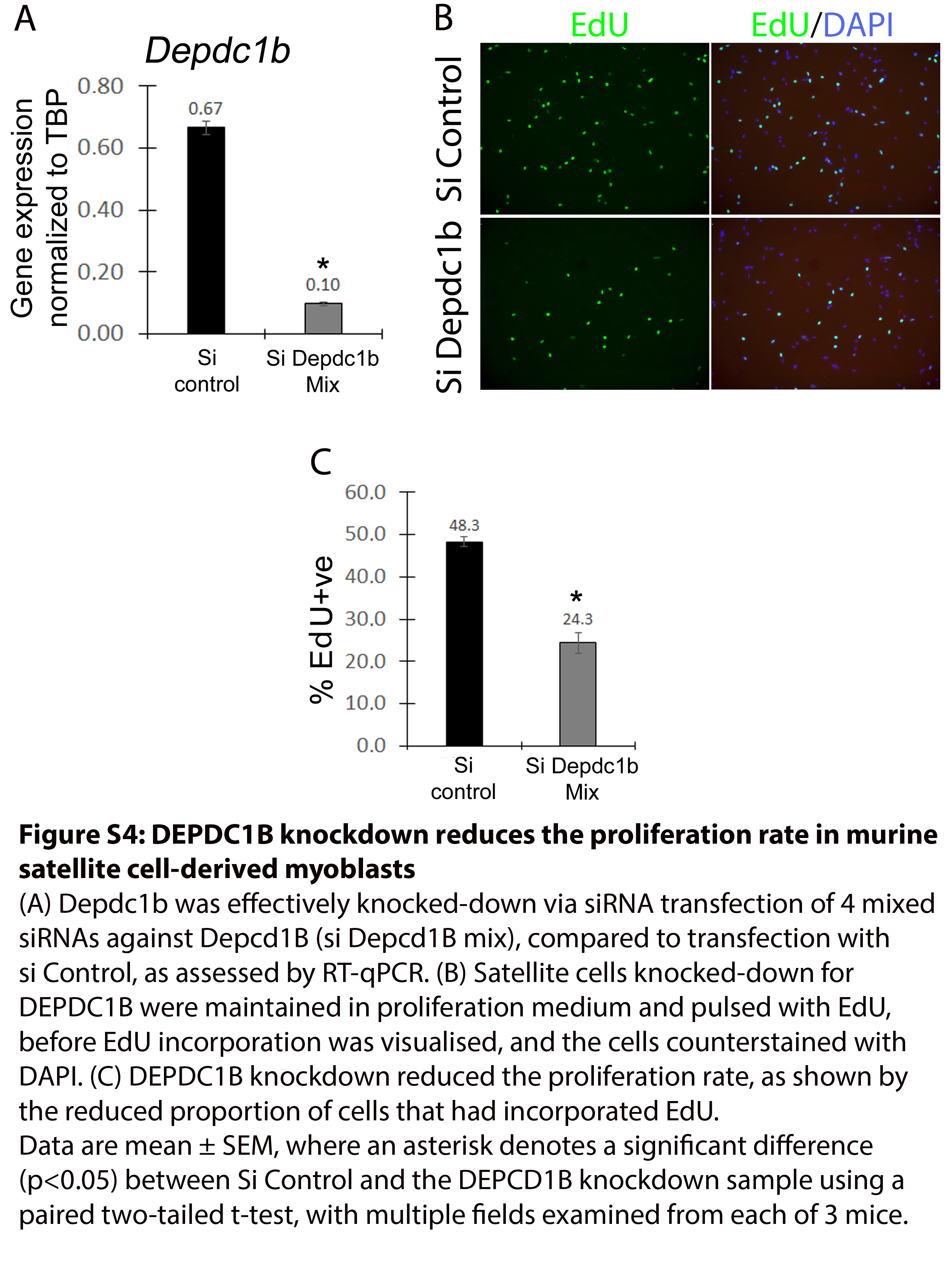

Supplement: Supplementary file 4 [file CPR-53-e12717-s004.jpg]

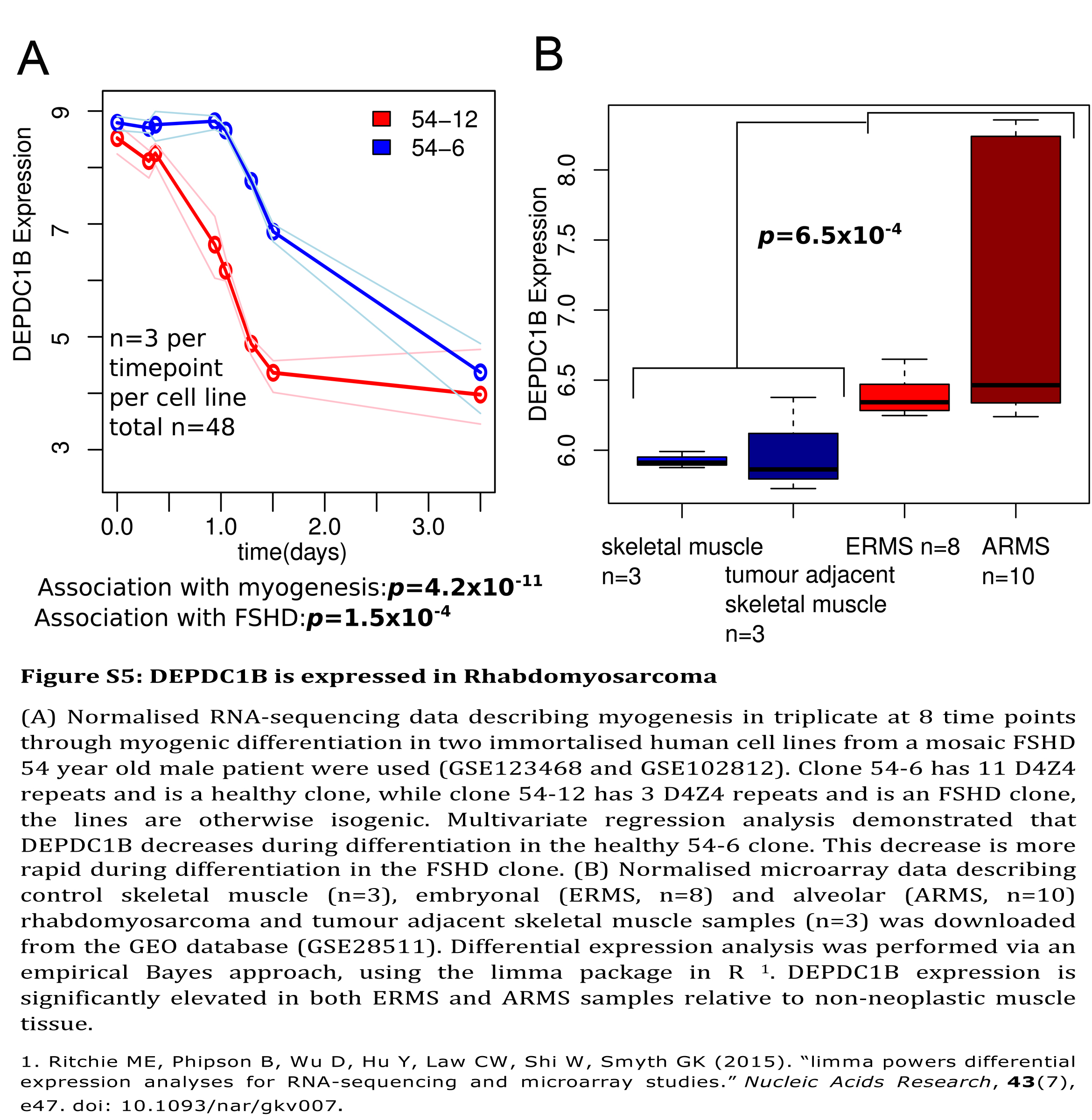

Supplement: Supplementary file 5 [file CPR-53-e12717-s005.jpg]

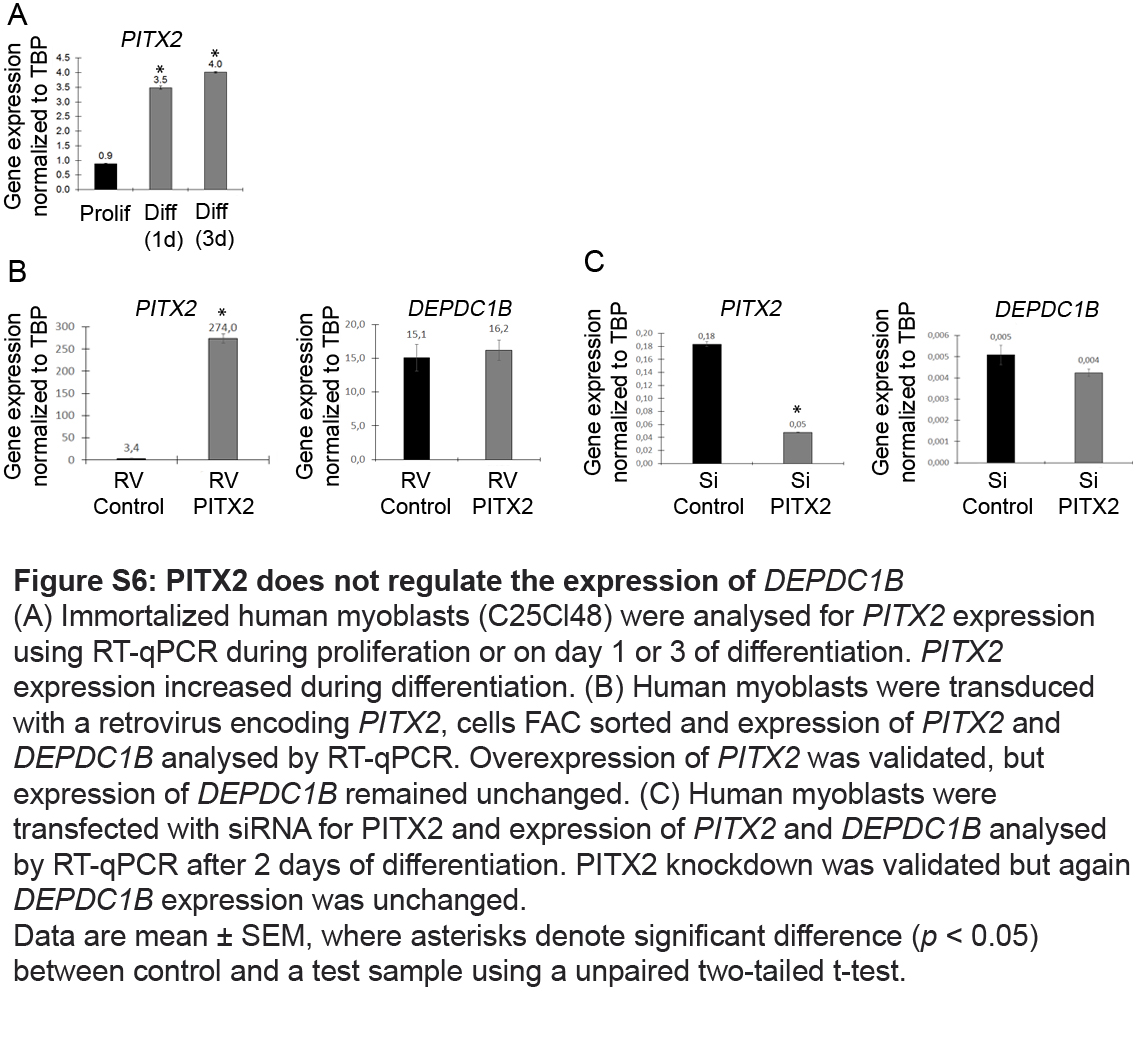

Supplement: Supplementary file 6 [file CPR-53-e12717-s006.jpg]

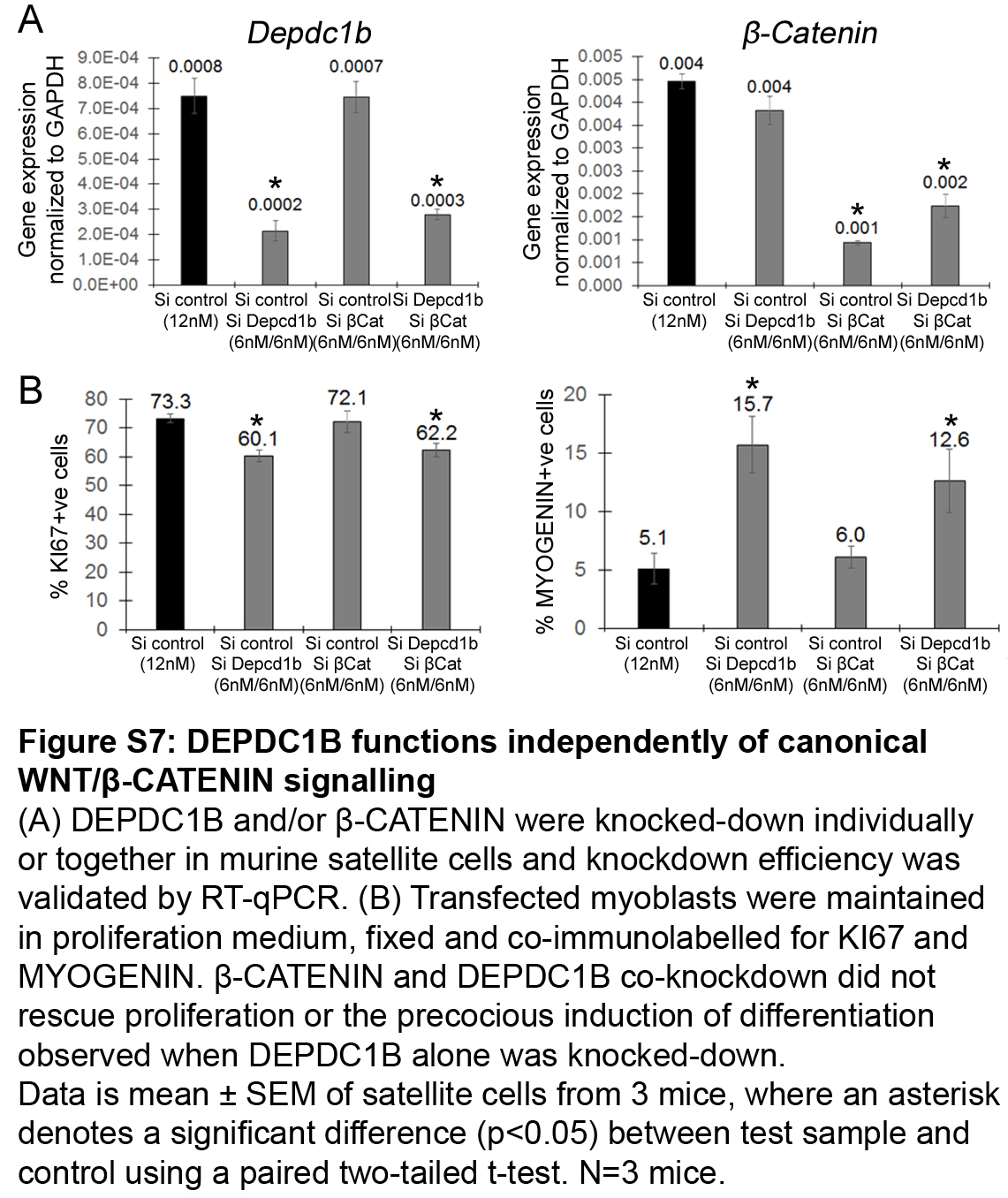

Supplement: Supplementary file 7 [file CPR-53-e12717-s007.jpg]
