# Supplementary material for: DEPDC1B is a key regulator of myoblast proliferation in mouse and man
Source: Cell Prolif. 2019 Dec 11;53(1):e12717. doi: 10.1111/cpr.12717 (PMC6985657; doi:10.1111/cpr.12717)
Supplement: Supplementary file 8 [file CPR-53-e12717-s008.docx]

**Supplementary Tables**

**Table S1: List of siRNAs**

| **siRNA** | **ID** | **Gene** | **Species** | **Company** | **Conc. (nM)** |
| --- | --- | --- | --- | --- | --- |
| Ambion: Silencer select pre-designed siRNA | s104323 | Depdc1b (N1) | Mus musculus | Life Technologies | 8/6/8 |
| Ambion: Silencer select pre-designed siRNA | s104324 | Depdc1b (N2) | Mus musculus | Life Technologies | 8 |
| GeneSolution siRNA | SI00977984 | Depdc1b | Mus musculus | QIAGEN | 2 |
| GeneSolution siRNA | SI00977977 | Depdc1b | Mus musculus | QIAGEN | 2 |
| GeneSolution siRNA | SI00977970 | Depdc1b | Mus musculus | QIAGEN | 2 |
| GeneSolution siRNA | SI00977963 | Depdc1b | Mus musculus | QIAGEN | 2 |
| GeneSolution siRNA | SI104230212 | Depdc1b | Homo sapiens | QIAGEN | 2 |
| GeneSolution siRNA | SI104322367 | Depdc1b | Homo sapiens | QIAGEN | 2 |
| GeneSolution siRNA | SI04341638 | Depdc1b | Homo sapiens | QIAGEN | 2 |
| GeneSolution siRNA | SI105150306 | Depdc1b | Homo sapiens | QIAGEN | 2 |
| Ambion: Silencer select pre-designed siRNA | s94095 | Depdc1a | Mus musculus | Life Technologies | 6 |
| Ambion: Silencer select pre-designed siRNA | s31138 | DEPDC1 | Homo sapiens | Life Technologies | 8 |
| Ambion: Silencer select pre-designed siRNA | s63417 | β-Catenin | Mus musculus | Life Technologies | 6 |
| GeneSolution siRNA | SI04342912 | PITX2 | Homo sapiens | QIAGEN | 2 |
| GeneSolution siRNA | SI04239711 | PITX2 | Homo sapiens | QIAGEN | 2 |
| GeneSolution siRNA | SI04149810 | PITX2 | Homo sapiens | QIAGEN | 2 |
| GeneSolution siRNA | SI00685279 | PITX2 | Homo sapiens | QIAGEN | 2 |
| GeneSolution siRNA | SI02776907 | RHOA | Homo sapiens | QIAGEN | 2 |
| GeneSolution siRNA | SI02654267 | RHOA | Homo sapiens | QIAGEN | 2 |
| GeneSolution siRNA | SI02654211 | RHOA | Homo sapiens | QIAGEN | 2 |
| GeneSolution siRNA | SI04434213 | RHOA | Homo sapiens | QIAGEN | 2 |
| GeneSolution siRNA | SI02658796 | PTRF | Homo sapiens | QIAGEN | 2 |
| GeneSolution siRNA | SI02658789 | PTRF | Homo sapiens | QIAGEN | 2 |
| GeneSolution siRNA | SI04026267 | PTRF | Homo sapiens | QIAGEN | 2 |
| GeneSolution siRNA | SI04026204 | PTRF | Homo sapiens | QIAGEN | 2 |
| Select Negative Control #2 siRNA | 4390847 |  |  | Life Technologies | 8-12-12 |
| AllStars Negative Control siRNA | SI03650318 |  |  | QIAGEN | 8-8-8-16-16-8 |

**Table S2: List of primers for RT-qPCR**

| **Gene** | **Species** | **Forward** | **Reverse** |
| --- | --- | --- | --- |
| ***Tbp*** | Mus musculus | ATCCCAAGCGATTTGCTG | CCTGTGCACACCATTTTTCC |
| ***Pax7*** | Mus musculus | CCGTGTTTCTCATGGTTGTG | GAGCACTCGGCTAATCGAAC |
| ***Depdc1a*** | Mus musculus | GGTGCTCCTCTTGGACCTTA | CATTCCTACTCGAAAAGATGTGG |
| ***Depdc1b*** | Mus musculus | CGTCAGCGTCTTAGGTTTGC | CCGGGAAAAGGTCTGAACCA |
| ***Ctnnb1* (β-CATENIN)** | Mus musculus | tgctgaaggtgctgtctgtc | TATCATCGGAACCCAGAAGC |
|  |  |  |  |
| **Gene** | **Species** | **Forward** | **Reverse** |
| ***TBP*** | Homo sapiens | cggctgtttaacttcgcttc | cacacgccaagaaacagtga |
| ***DEPDC1A*** | Homo sapiens | CGCTGACAGACCTATGGAGAG | CCTGCTGGAAAAGATGTGGT |
| ***DEPDC1B*** | Homo sapiens | TGGTACCCGAACACTGATGG | GACGGCAAAATGATGGAGCA |
| ***PTPRF*** | Homo sapiens | cctgcgaacctgtatgtgc | catcacctcctggctgct |
|  |  |  |  |
| ***MYF5*** | Homo sapiens | CTATAGCCTGCCGGGACA | TGGACCAGACAGGACTGTTACAT |
| ***PAX3*** | Homo sapiens | AGGAGGCCGACTTGGAGA | CTTCATCTGATTGGGGTGCT |
| ***MYOD1*** | Homo sapiens | cactacagcggcgactcc | taggcgccttcgtagcag |
| ***MYOGENIN*** | Homo sapiens | CCAGGGGTGCCCAGCGAATG | AGCCGTGAGCAGATGATCC |
| ***PITX2*** | Homo sapiens | Ctgtgtggaccaaccttacg | Ccgaagccattcttgcata |
|  |  |  |  |
| ***RHOA*** | Homo sapiens | gggagctagccaagatgaag | gtacccaaaagcgccaatc |
|  |  |  |  |
| ***RB1*** | Homo sapiens | Cttcctcatgctgttcaggag | tgcatgaagaccgagttatagaat |
| ***E2F1*** | Homo sapiens | Aagtccaagaaccacatccagt | ctgggtcaacccctcaag |
| ***CDC6*** | Homo sapiens | tgctcttgatcaggcagttg | tgcgacagactttactgtaggc |
| ***ORC1*** | Homo sapiens | aaggcctttgaagatgatgc | catctccagacagtgctgcta |
| ***CENPF*** | Homo sapiens | gagtcctccaaaccaacagc | tccgctgagcaactttgac |
|  |  |  |  |
| ***CCNA1* (CYCLIN A1)** | Homo sapiens | aaatgggcagtacaggagga | ccacagtcagggagtgcttt |
| ***CCNA2* (CYCLIN A2)** | Homo sapiens | ggtactgaagtccgggaacc | gaagatccttaaggggtgcaa |
| ***CCNB1* (CYCLIN B1)** | Homo sapiens | catggtgcactttcctcctt | aggtaatgttgtagagttggtgtcc |
| ***CCNB2* (CYCLIN B2)** | Homo sapiens | tggaaaagttggctccaaag | tcagaaaaagcttggcagaga |
| ***CCND1* (CYCLIN D1)** | Homo sapiens | gctgtgcatctacaccgaca | ttgagcttgttcaccaggag |
| ***CCND2* (CYCLIN D2)** | Homo sapiens | ggacatccaaccctacatgc | cgcacttctgttcctcacag |
| ***CCND3* (CYCLIN D3)** | Homo sapiens | gcttactggatgctggaggta | aagacaggtagcgatccaggt |
| ***CCNE1* (CYCLIN E1)** | Homo sapiens | ggccaaaatcgacaggac | gggtctgcacagactgcat |
|  |  |  |  |
| ***CDK1*** | Homo sapiens | tggatctgaagaaatacttggattcta | caatcccctgtaggatttgg |
| ***CDK2*** | Homo sapiens | aaagccagaaacaagttgacg | gtactgggcacaccctcagt |
| ***CDK4*** | Homo sapiens | gtgcagtcggtggtacctg | aggcagagattcgcttgtgt |
| ***CDK6*** | Homo sapiens | tgatcaactaggaaaaatcttggac | ggcaacatctctaggccagt |
|  |  |  |  |
| ***CDKN1A* (P21)** | Homo sapiens | ccgaagtcagttccttgtgg | catgggttctgacggacat |
| ***CDKN1B* (P27)** | Homo sapiens | tttgacttgcatgaagagaagc | agctgtctctgaaagggacatt |
| ***CDKN1C* (P57)** | Homo sapiens | ctcctttccccttcttctcg | tccatcgtggatgtgctg |
| ***CDKN2A* (P16)** | Homo sapiens | cacattcatgtgggcatttc | tgcttgtcatgaagtcgacag |
| ***CDKN2B* (P15)** | Homo sapiens | caacggagtcaaccgtttc | ggtgagagtggcagggtct |
| ***CDKN2C* (P18)** | Homo sapiens | gactatcccttcggcgaga | aaggctcggccattctttag |
| ***CDKN2D* (P19)** | Homo sapiens | tgcaggtcatgatgtttgg | gctggcaccttgcttcag |
| ***TP53*** | Homo sapiens | Gcatgtctgtctatgctgtgc | ttcattttgagcttccactctg |

**Table S3: List of antibodies and Phalloidin**

| **Protein** | **Host** | **Dilution** | **Clonality** | **Reference** |
| --- | --- | --- | --- | --- |
| **DEPDC1B** | Rabbit | 1/100 | Polyclonal | HPA038255 (Sigma) |
| **MYOGENIN** | Mouse | 1/10 | Monoclonal | F5D (DSHB) |
| **MyHC** | Mouse | 1/300 | Monoclonal | MF20c (DSHB) |
| **β-Tubulin** | Mouse | 1/300 | Monoclonal | E7c (DSHB) |
| **GFP** | Chicken | 1/2000 | Polyclonal | ab13970 (abcam ) |
| **β-CATENIN** | Mouse | 1/200 | Monoclonal | 610154 (BD transduction laboratories) |
| **V5 epitope Tag** | Rabbit | 1/500 | Polyclonal | AB3792 (MILLIPORE) |
| **KI67** | Rabbit | 1/500 | Monoclonal | ab16667 (abcam) |
| **Alexa Fluor™ 594 Phalloidin** |  | 1/40 |  | A12381 (ThermoFisher scientific) |
